# Hsa_circ_0003204 Knockdown Weakens Ox-LDL-Induced Cell Injury by Regulating miR-188-3p/TRPC6 Axis in Human Carotid Artery Endothelial Cells and THP-1 Cells

**DOI:** 10.3389/fcvm.2021.731890

**Published:** 2021-11-29

**Authors:** Wenjia Peng, Shuai Li, Shiyue Chen, Jiacheng Yang, Ze Sun

**Affiliations:** Department of Radiology, The First Affiliated Hospital of Naval Medical University, Shanghai, China

**Keywords:** carotid artery AS, ox-LDL, circ_0003204, MiR-188-3p, TRPC6

## Abstract

**Background:** Circular RNAs (circRNAs) are involved in atherosclerosis (AS) development. However, the function and mechanism of circRNA hsa_circ_0003204 (circ_0003204) in carotid artery AS remain unclear.

**Methods:** Oxidized low-density lipoprotein (ox-LDL)-treated human carotid artery endothelial cells (HCtAECs) and THP-1 cells were used as cell models of carotid artery AS. Relative levels of circ_0003204, microRNA-188-3p (miR-188-3p), and transient receptor potential canonical channel 6 (TRPC6) were detected by quantitative reverse transcription–polymerase chain reaction or Western blotting. The targeting relationship between circ_0003204 or TRPC6 and miR-188-3p was assessed via dual-luciferase reporter analysis and RNA immunoprecipitation. Cell proliferation was assessed via 3-(4,5-dimethyl-2-thiazolyl)-2,5-diphenyl-2*H*-tetrazolium bromide assay and 5-ethynyl-2′-deoxyuridine (EdU) assay. Cell apoptosis was analyzed via assessing cell caspase-3 activity, apoptosis, and apoptosis-related protein. Inflammatory response was analyzed via analysis of interleukin-1β (IL-1β), IL-6, and tumor necrosis factor-α (TNF-α). Oxidative stress was assessed via determination of reactive oxygen species (ROS), malondialdehyde (MDA), and superoxide dismutase (SOD).

**Results:** Circ_0003204 and TRPC6 levels were elevated, and miR-188-3p expression declined in ox-LDL-treated HCtAECs and THP-1 cells. Circ_0003204 could regulate TRPC6 expression via mediating miR-188-3p. Circ_0003204 silencing weakened ox-LDL-induced viability inhibition and apoptosis in HCtAECs, and inflammatory response and oxidative stress in THP-1 cells via regulating miR-188-3p. MiR-188-3p overexpression attenuated ox-LDL-induced injury in HCtAECs and THP-1 cells by targeting TRPC6.

**Conclusion:** Circ_0003204 knockdown mitigated ox-LDL-induced injury in HCtAECs and THP-1 cells via regulating the miR-188-3p/TRPC6 axis, indicating that circ_0003204 might play an important role in carotid artery AS.

## Introduction

Atherosclerosis (AS) is an inflammatory-related cardiovascular disease (1). Carotid artery AS is a group of AS and associated with the increased risk of cardiovascular disorders (2). Oxidized low-density lipoprotein (ox-LDL) has an essential role in AS development via regulating the function of multiple cell lines, like endothelial cells and macrophages (3). The malfunction of endothelial and THP-1 cells is implicated in the pathobiology of AS (4, 5). Therefore, analyzing the pathogenesis of ox-LDL-triggered dysfunction of carotid artery endothelial cells and THP-1 cells may help to explore new strategies for carotid artery AS treatment.

Circular RNAs (circRNAs) and microRNAs (miRNAs) are correlated with the regulation of cardiovascular cell biology in AS (6). CircRNAs are stable ncRNAs formed via back-splicing events, which act as vital biomarkers for cardiovascular diseases, including AS (7). CircRNA hsa_circ_0003204 (circ_0003204), derived from ubiquitin-specific peptidase 36 (USP36), has been reported to be upregulated in ox-LDL-challenged human aortic endothelial cells (HAECs) and plays a vital role in cerebrovascular atherogenesis progression (8). Moreover, circ_0003204 is reported to be dysregulated in ox-LDL-irritated human umbilical vein endothelial cells (HUVECs) (9). Hence, we assumed that circ_0003204 might play a vital role in AS progression. However, how and whether circ_0003204 takes part in the development of carotid artery AS remain unknown.

MiRNAs have been suggested to participate in AS development (10). According to the ceRNA hypothesis, circRNAs can regulate gene expression via binding to miRNAs (11). In this study, we screened the top 10 miRNAs (miR-1224-3p, miR-1236, miR-346, miR-370, miR-432, miR-593, miR-635, miR-1827, miR-620, and miR-188-3p) based on context + score percentage after circular RNA Interactome prediction. And preliminary experiments exhibited that miR-188-3p was highly pulled down by circ_0003204 probe. A previous study indicated that miR-188-3p could inhibit the inflammatory response in AS mice (12). However, whether miR-188-3p is involved in circ_0003204-mediated carotid artery AS development is unclear.

The transient receptor potential canonical channels (TRPCs) play an important role in cardiovascular diseases (13). Among all miR-188-3p targets predicted by the DIANA tool, five mRNAs [KLF6, IGF2, AKT3, TRIM14, and TRPC 6 (TRPC6)] have been reported to have the opposite function of miR-188-3p. And preliminary experiments showed that TRPC6 was pulled down the most by the miR-188-3p probe. TRPC6, a key member of TRPCs, contributes to ox-LDL-induced HAEC apoptosis (14). At present, the involvement of circ_0003204, miR-188-3p, and TRPC6 in carotid artery AS is unclear. Hence, we hypothesized that circ_0003204 might regulate carotid artery AS development via mediating miR-188-3p/TRPC6 axis.

In this research, we utilized ox-LDL-challenged HCtAECs and THP-1 cells to mimic carotid artery AS environment (15). Moreover, we detected the expression of circ_0003204, miR-188-3p, and TRPC6, and we explored the function of circ_0003204 in ox-LDL-triggered cell injury. Additionally, we explored the ceRNA network of circ_0003204/miR-188-3p/TRPC6 axis.

## Materials and Methods

### Cell Culture and Treatment

HCtAECs were provided via Cell Applications (San Diego, CA, USA) and cultured in specific MesoEndo Cell Grown Medium (Cell Applications) in 5% CO_2_ at 37°C. THP-1 cells were offered by Procell (Wuhan, China) and maintained in RPMI-1640 medium (Procell) containing 10% fetal bovine serum (FBS) (HyClone, Logan, UT, USA) and 1% penicillin/streptomycin (Thermo Fisher, Waltham, MA, USA) in 5% CO_2_ at 37°C.

To mimic the carotid AS-like microenvironment, HCtAECs and THP-1 cells were challenged via different doses of ox-LDL (Solarbio, Beijing, China) for 24 h.

### Real-Time Quantitative PCR

The RNA was extracted by TRIzol (Thermo Fisher) (16). The RNA was reversely transcribed using a specific reverse transcription kit (Thermo Fisher). The cDNA was mixed with SYBR (Vazyme, Nanjing, China) and specific primers (Genscript, Nanjing, China) and used for qRT-PCR. The primers were as follows: circ_0003204 (F, 5′-CTCAAATGCCCAAGGAGTGC-3′; R, 5′-GCAGGCGGCTGGATGATT-3′), TRPC6 (F, 5′-AGGGCTGGAGAGTCTCTGTT-3′; R, 5′-TGGTGGTAGCGAAGCGTAAG-3′), miR-188-3p (F, 5′-CTCCCACATGCAGGG-3′; R, 5′-GTGCAGGGTCCGAGGT-3′), U6 (F, 5′-CTCGCTTCGGCAGCACA-3′; R, 5′-AACGCTTCACGAATTTGCGT-3′), and GAPDH (F, 5′-GAATGGGCAGCCGTTAGGAA-3′; R, 5′-AAAAGCATCACCCGGAGGAG-3′). U6 or GAPDH served as a reference control. RNA level was computed using the 2^−ΔΔCt^ method (17).

### Western Blotting

Cells were lysed using RNA immunoprecipitation (RIPA) (Solarbio), and protein samples were collected via centrifugation. Proteins of 20 μg were loaded on sodium dodecyl sulfate–polyacrylamide gel electrophoresis (SDS-PAGE) and transferred to nitrocellulose membranes (Solarbio). The membranes were blocked in 5% fat-free milk and incubated with primary antibodies anti-TRPC6 (ab62461, Abcam, Cambridge, MA, USA), anti-BAX (ab104156, Abcam), anti-BCL2 (ab194583, Abcam), or anti-β-actin (ab8227, Abcam) and the secondary antibody (ab205718, Abcam). Next, the bands were exposed to enhanced chemiluminescence (ECL) reagent (Solarbio).

### Dual-Luciferase Reporter Analysis and RNA Immunoprecipitation

The wild-type luciferase reporter plasmid circ_0003204-WT was constructed via cloning the wild-type sequence of circ_0003204 into psiCHECK-2 vectors (YouBio, Changsha, China). HCtAECs and THP-1 cells were co-transfected with circ_0003204-WT, circ_0003204-WT+miR-con, circ_0003204-WT+miR-188-3p mimic, circ_0003204-WT+miR-188-3p mimic+pcDNA, or circ_0003204-WT+miR-188-3p mimic+TRPC6 overexpression vector for 24 h. Next, the luciferase intensity was analyzed via a dual-luciferase analysis kit.

The Magna RIP Kit (Sigma, St. Louis, MO, USA) was exploited for RIP analysis; 1 × 10^7^ HCtAECs and THP-1 cells were lysed and interacted with anti-Ago2 or anti-IgG-conjugated magnetic beads for 6 h. The enrichment of circ_0003204, miR-188-3p, and TRPC6 in the complex was detected by qRT-PCR.

### Cell Transfection

Circ_0003204 overexpression vector was synthesized by cloning circ_0003204 sequence into pcDNA3.1 circRNA mini vector, with the pcDNA3.1 circRNA mini vector (Addgene, Cambridge, MA, USA) as negative control (vector). TRPC6 overexpression vector was generated via inserting the full length of TRPC6 (accession: NM_004621.6) sequence into pcDNA3.1 vector, with the pcDNA3.1 vector (Addgene) as negative control (pcDNA). SiRNA for circ_0003204 (si-circ_0003204, 5′-CCGCAUGGGGCUGUGUCACCU-3′), negative control of siRNA (si-con, 5′-AAGACAUUGUGUGUCCGCCTT-3′), miR-188-3p mimic (5′-CUCCCACAUGCAGGGUUUGCA-3′), negative control of mimic (miR-con, 5′-ACGUGACACGUUCGGAGAATT-3′), miR-188-3p inhibitor (anti-miR-188-3p, 5′-UGCAAACCGACUUGUGGGAG-3′), and negative control of inhibitors (anti-miR-con, 5′-UGAGCUGCAUAGAGUAGUGAUUA-3′) were synthesized via RiboBio (Guangzhou, China). HCtAECs and THP-1 cells were transfected using Lipofectamine 2000 (Thermo Fisher).

### Cell Viability

Cell viability was tested by 3-(4,5-dimethyl-2-thiazolyl)-2,5-diphenyl-2*H*-tetrazolium bromide (MTT) analysis; 1 × 10^4^ HCtAECs were added into 96-well plates overnight and stimulated via 100 μg/ml of ox-LDL for 24 h. Next, culture medium was changed to fresh one plus 0.1 mg/ml MTT (Solarbio). After culture for 4 h, the medium was removed, and each well was added with 100 μl of dimethyl sulfoxide (DMSO) (Beyotime, Shanghai, China). The absorbance was examined at 570 nm using a microplate reader.

### 5-Ethynyl-2′-Deoxyuridine Assay

After relevant transfection and ox-LDL treatment, 5-ethynyl-2′-deoxyuridine (EdU) assay kit (Beyotime) was used for cell proliferation. In brief, cells were seeded into 24-well plates (5 × 10^3^ cells/well), and then EdU was added for 2 h of incubation. After that, the cells were fixed with 4% paraformaldehyde (Sigma) and mixed with 0.5% Triton X-100 (Sigma), followed by incubation with Apollo and DAPI. Last, EDU-positive cells were quantified.

### Caspase-3 Activity and Flow Cytometry

For detection of caspase-3 activity, 4 × 10^5^ HCtAECs were added into 6-well plates and exposed to 100 μg/ml of ox-LDL. Next, cells were lysed for caspase-3 activity analysis using a caspase-3 assay kit (Abcam) according to the instruction of the manufacturer.

For analysis of cell apoptotic rate, 2 × 10^5^ HCtAECs were placed into 6-well plates overnight and then stimulated via 100 μg/ml of ox-LDL for 24 h. Next, cells were detected using Annexin V-FITC apoptosis detection kit (Sigma). The apoptotic cells were examined using a flow cytometer (Agilent, Hangzhou, China).

### Enzyme-Linked Immunosorbent Assay

The inflammatory response was assessed via analysis of the levels of IL-1β, IL-6, and TNF-α; 1 × 10^5^ THP-1 cells were placed into 12-well plates overnight and then treated via 100 μg/ml of ox-LDL for 24 h. Next, the medium was collected and used for analysis of IL-1β, IL-6, and TNF-α levels using specific ELISA kits (Thermo Fisher) following the instructions of the manufacturer.

### Detection of Malondialdehyde, Superoxide Dismutase, and Reactive Oxygen Species Levels

The oxidative stress was analyzed by evaluation of malondialdehyde (MDA), superoxide dismutase (SOD), and reactive oxygen species (ROS) levels; 4 × 10^4^ THP-1 cells were placed into 24-well plates overnight and then challenged via 100 μg/ml of ox-LDL for 24 h. Next, cells were lysed; and the lysis solution was used for analysis of MDA and SOD levels using MDA or SOD Assay kit (Sigma) following the instructions of the manufacturer. The levels of MDA and SOD were normalized to total protein in cells. In addition, 2 × 10^4^ THP-1 cells were added into 96-well plates overnight and then treated via 100 μg/ml of ox-LDL for 24 h. Next, cells were analyzed using a cellular ROS assay kit (Abcam). The ROS level was normalized to the control group (not treated with ox-LDL).

### Statistical Analysis

The experiments were performed 3 times × 3 replicates. The data were presented as mean ± SD. The difference was compared via Student's *t*-test or ANOVA with Dunnett's test using SPSS 20.0. *p* < 0.05 indicated the statistical significance.

## Results

### Circ_0003204 and Transient Receptor Potential Canonical Channel 6 Levels Are Increased and microRNA-188-3p Expression Is Decreased in Oxidized Low-Density Lipoprotein-Treated Human Carotid Artery Endothelial Cells and THP-1 Cells

**Figure 1:**
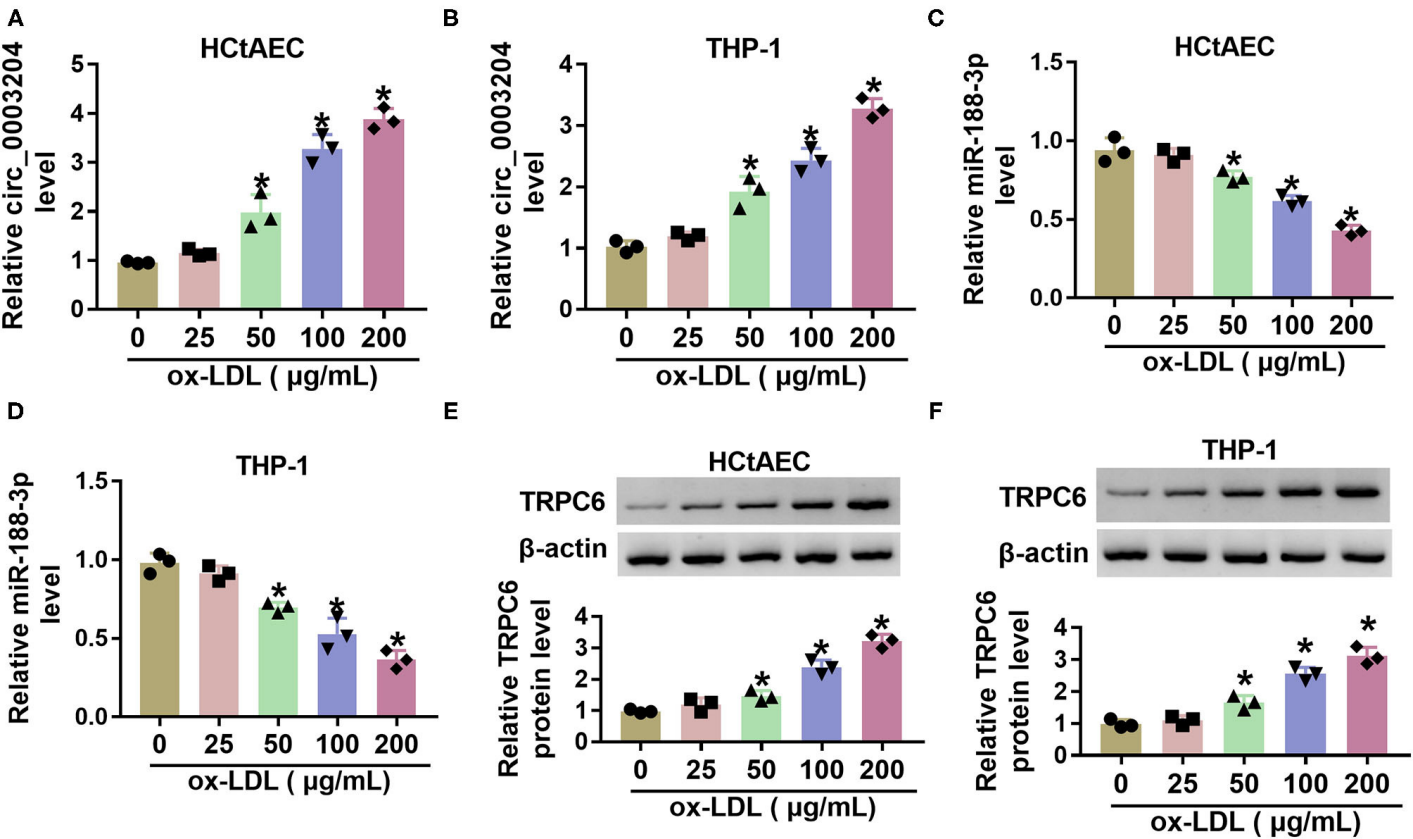
The levels of circ_0003204, miR-188-3p, and TRPC6 in ox-LDL-treated HCtAECs and THP-1 cells. Circ_0003204 expression **(A,B)** (one-way ANOVA), miR-188-3p expression **(C,D)** (one-way ANOVA), and TRPC6 protein level **(E,F)** (one-way ANOVA) were detected in HCtAECs and THP-1 cells after exposure to various doses of ox-LDL for 24 h. *p < 0.05. TRPC6, transient receptor potential canonical channel 6; ox-LDL, oxidized low-density lipoprotein; HCtAECs, human carotid artery endothelial cells.

To explore whether circ_0003204, miR-188-3p, and TRPC are implicated in carotid AS, their levels were detected in ox-LDL-challenged HCtAECs and THP-1 cells. Circ_0003204 expression was evidently increased in ox-LDL-treated HCtAECs and THP-1 cells in a dose-dependent manner (Figures 1A,B). Furthermore, miR-188-3p level was progressively reduced as the elevation of ox-LDL (Figures 1C,D). Additionally, TRPC6 protein expression was markedly elevated in the two cell lines after exposure to different doses of ox-LDL (Figures 1E,F). These data indicated that circ_0003204, miR-188-3p, and TRPC might be associated with ox-LDL-induced injury.

### Circ_0003204 Regulates Transient Receptor Potential Canonical Channel Expression via Mediating microRNA-188-3p

**Figure 2:**
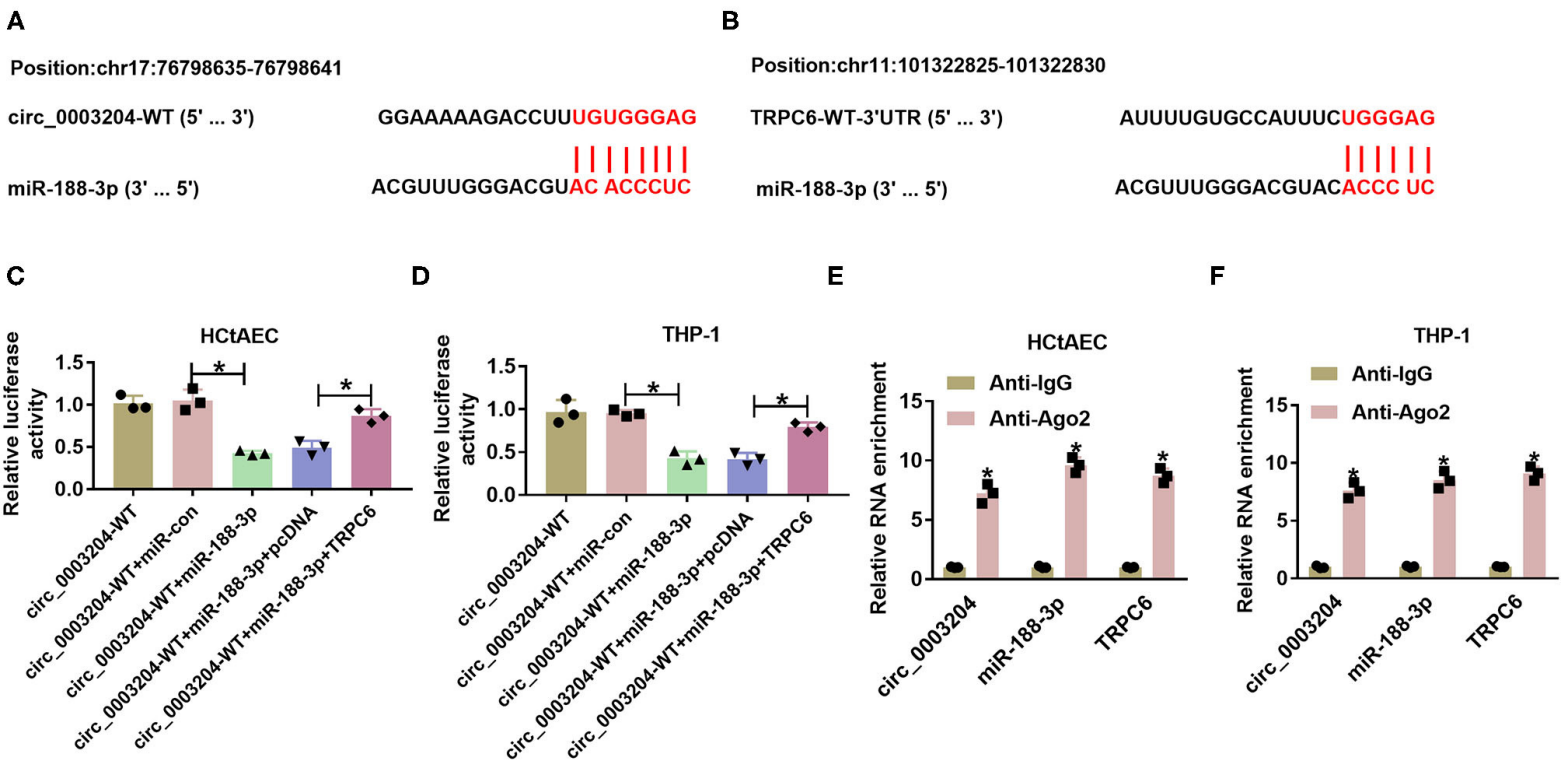
The target relationship between miR-188-3p and circ_0003204 or TRPC6. **(A,B)** The binding sequence between miR-188-3p and circ_0003204 was explored via Circular RNA Interactome, and that between miR-188-3p and TRPC6 was searched via DIANA tool. **(C,D)** Luciferase activity was detected in HCtAECs and THP-1 cells transfected with circ_0003204-WT, circ_0003204-WT+miR-con, circ_0003204-WT+miR-188-3p mimic, circ_0003204-WT+miR-188-3p mimic+pcDNA, or circ_0003204-WT+miR-188-3p mimic+TRPC6 overexpression vector (one-way ANOVA). **(E,F)** circ_0003204, miR-188-3p, and TRPC6 levels were detected in HCtAECs and THP-1 cells after Ago2 or IgG RIP (two-way ANOVA). *p < 0.05. TRPC6, transient receptor potential canonical channel 6; HCtAECs, human carotid artery endothelial cells.

**Figure 3:**
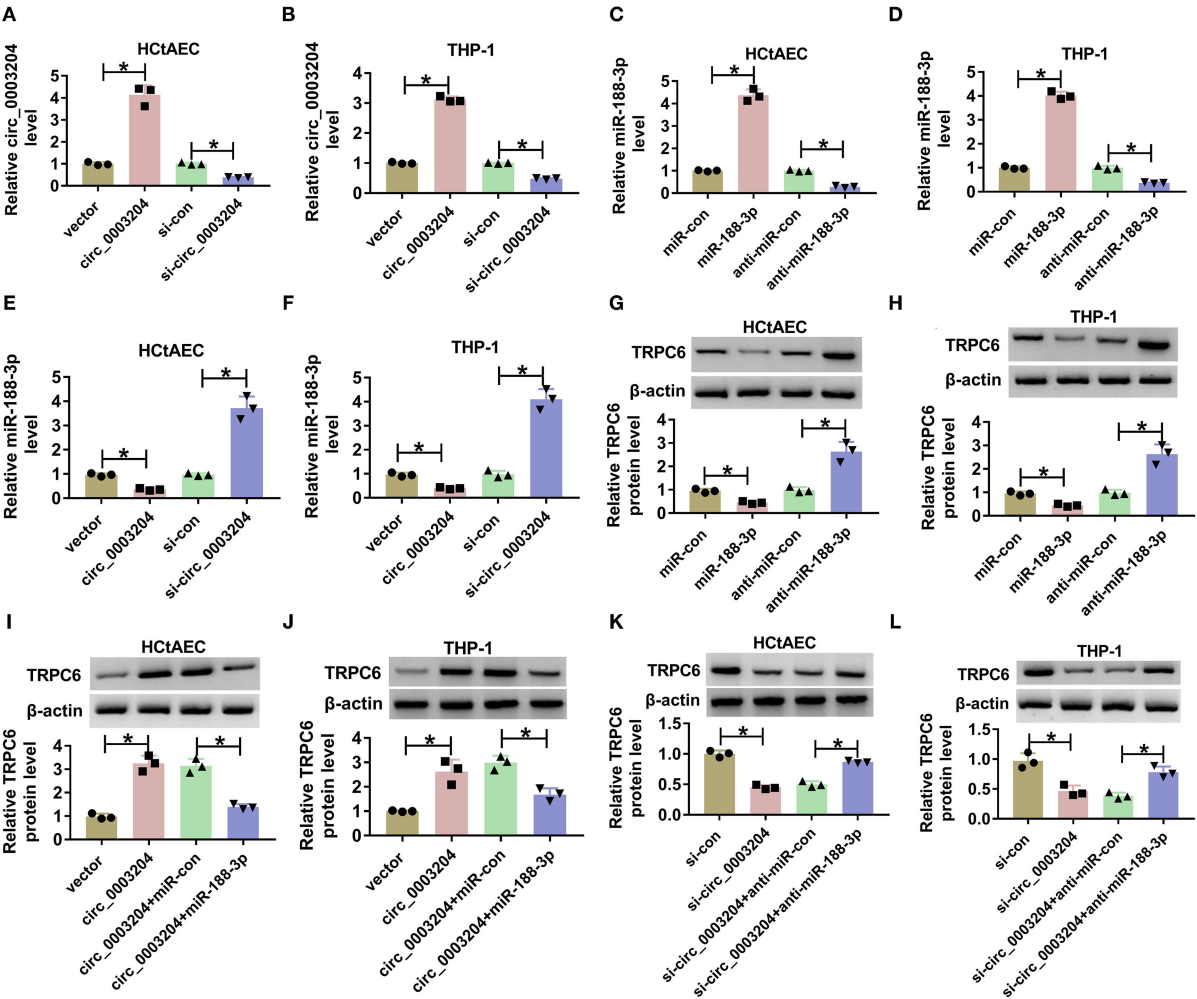
The regulatory effect of circ_0003204 on miR-188-3p and TRPC6. **(A,B)** The expression of circ_0003204 in HCtAECs and THP-1 cells transfected with vector, circ_0003204, si-con, or si-circ_00003204 was detected (one-way ANOVA). **(C,D)** The expression of miR-188-3p in miR-con, miR-188-3p, anti-miR-con, or anti-miR-188-3p transfected HCtAECs and THP-1 cells was detected (one-way ANOVA). **(E,F)** MiR-188-3p expression was detected in HCtAECs and THP-1 cells with transfection of vector, circ_0003204 overexpression vector, si-con, or si-circ_0003204 (one-way ANOVA). **(G,H)** TRPC6 protein expression was measured in HCtAECs and THP-1 cells with transfection of miR-con, miR-188-3p mimic, anti-miR-con, or anti-miR-188-3p (one-way ANOVA). **(I–L)** TRPC6 protein level was examined in HCtAECs and THP-1 cells transfected with vector, circ_0003204 overexpression vector, circ_0003204 overexpression vector+miR-con or miR-188-3p mimic **(I,J)** (one-way ANOVA), si-con, si-circ_0003204, or si-circ_0003204+anti-miR-con or anti-miR-188-3p **(K,L)** (one-way ANOVA). *p < 0.05. TRPC6, transient receptor potential canonical channel 6; HCtAECs, human carotid artery endothelial cells.

We further analyzed whether circ_0003204 could act as a ceRNA to regulate the miR-188-3p/TRPC axis. The predicted binding sequences between miR-188-3p and circ_0003204 or TRPC are displayed in Figures 2A,B. To identify their interactions, the dual-luciferase reporter analysis was performed in HCtAECs and THP-1 cells transfected with circ_0003204-WT, circ_0003204-WT+miR-con, circ_0003204-WT+miR-188-3p mimic, circ_0003204-WT+miR-188-3p mimic+pcDNA, or TRPC overexpression vector. The luciferase activity of circ_0003204-WT evidently declined via miR-188-3p overexpression, which was impaired via introduction of TRPC6 (Figures 2C,D). Furthermore, the RIP analysis using anti-Ago2 revealed that circ_0003204, miR-188-3p, and TRPC were enriched in the same complex (Figures 2E,F). As exhibited in Figures 3A,B, transfection of the circ_0003204 overexpression vector led to an elevation in circ_0003204 level, whereas transfection of si-circ_0003204 led to a reduction in circ_0003204 level in both HCtAECs and THP-1 cells. Transfection of MiR-188-3p mimic increased miR-188-3p expression, but introduction of anti-miR-188-3p reduced miR-188-3p expression in both HCtAECs and THP-1 cells (Figures 3C,D). Additionally, miR-188-3p expression was significantly reduced via circ_0003204 overexpression and enhanced via circ_0003204 knockdown (Figures 3E,F). Moreover, TRPC6 protein level was negatively regulated by miR-188-3p in HCtAECs and THP-1 cells (Figures 3G,H). As expected, TRPC protein expression was increased by circ_0003204 overexpression, whereas this elevation was reversed after miR-188-3p overexpression (Figures 3I,J). In contrast, circ_0003204 silencing resulted in a decrease in TRPC protein levels in HCtAECs and THP-1 cells, but this reduction was weakened after miR-188-3p inhibition (Figures 3K,L). These data indicated that circ_0003204 could function as a ceRNA for miR-188-3p to modulate TRPC6.

### Circ_0003204 Knockdown Attenuates Oxidized Low-Density Lipoprotein-Induced Injury via Regulating microRNA-188-3p in Human Carotid Artery Endothelial Cells and THP-1 Cells

**Figure 4:**
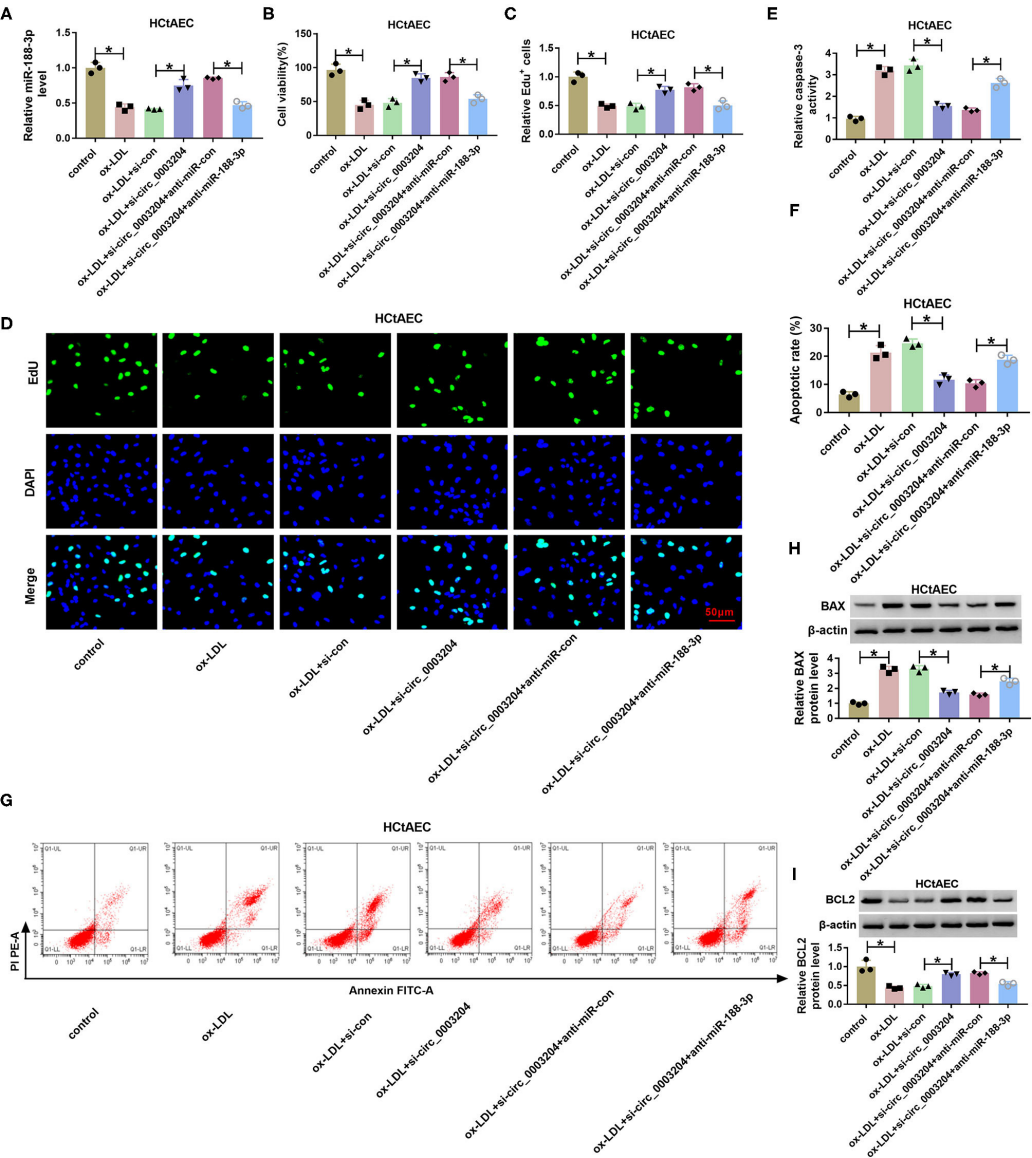
The effect of circ_0003204 and miR-188-3p on HCtAEC viability and apoptosis. HCtAECs were transfected with si-con, si-circ_0003204, si-circ_0003204+anti-miR-con, or anti-miR-188-3p before treatment of ox-LDL. **(A)** The expression of miR-188-3p in HCtAECs was detected (one-way ANOVA). **(B–D)** Cell viability and proliferation were assessed by MTT assay and EdU assay (one-way ANOVA). **(E)** Caspase-3 activity was detected (one-way ANOVA). **(F,G)** The apoptosis of HCtAECs was analyzed by flow cytometry analysis (one-way ANOVA). **(H,I)** The protein levels of BAX and BCL2 in HCtAECs were measured (one-way ANOVA). *p < 0.05. HCtAEC, human carotid artery endothelial cell; ox-LDL, oxidized low-density lipoprotein; EdU, 5-ethynyl-2′-deoxyuridine.

To probe into whether circ_0003204 mediated ox-LDL-induced HCtAEC injury and through miR-188-3p, HCtAECs were transfected with si-con, si-circ_0003204, si-circ_0003204+anti-miR-con, or anti-miR-188-3p before treatment of ox-LDL. qRT-PCR assay showed that circ_0003204 knockdown increased miR-188-3p level in ox-LDL-induced HCtAECs, while introduction of anti-miR-188-3p reversed the effect (Figure 4A). As displayed in Figures 4B–D, circ_0003204 knockdown mitigated ox-LDL-induced viability and proliferation inhibition in HCtAECs, which was weakened by miR-188-3p inhibition. Moreover, circ_0003204 silencing weakened ox-LDL-induced apoptosis by decreasing caspase-3 activity and regulating BAX and BCL2 protein levels in HCtAECs, which was abolished via miR-188-3p downregulation (Figures 4E–I).

**Figure 5:**
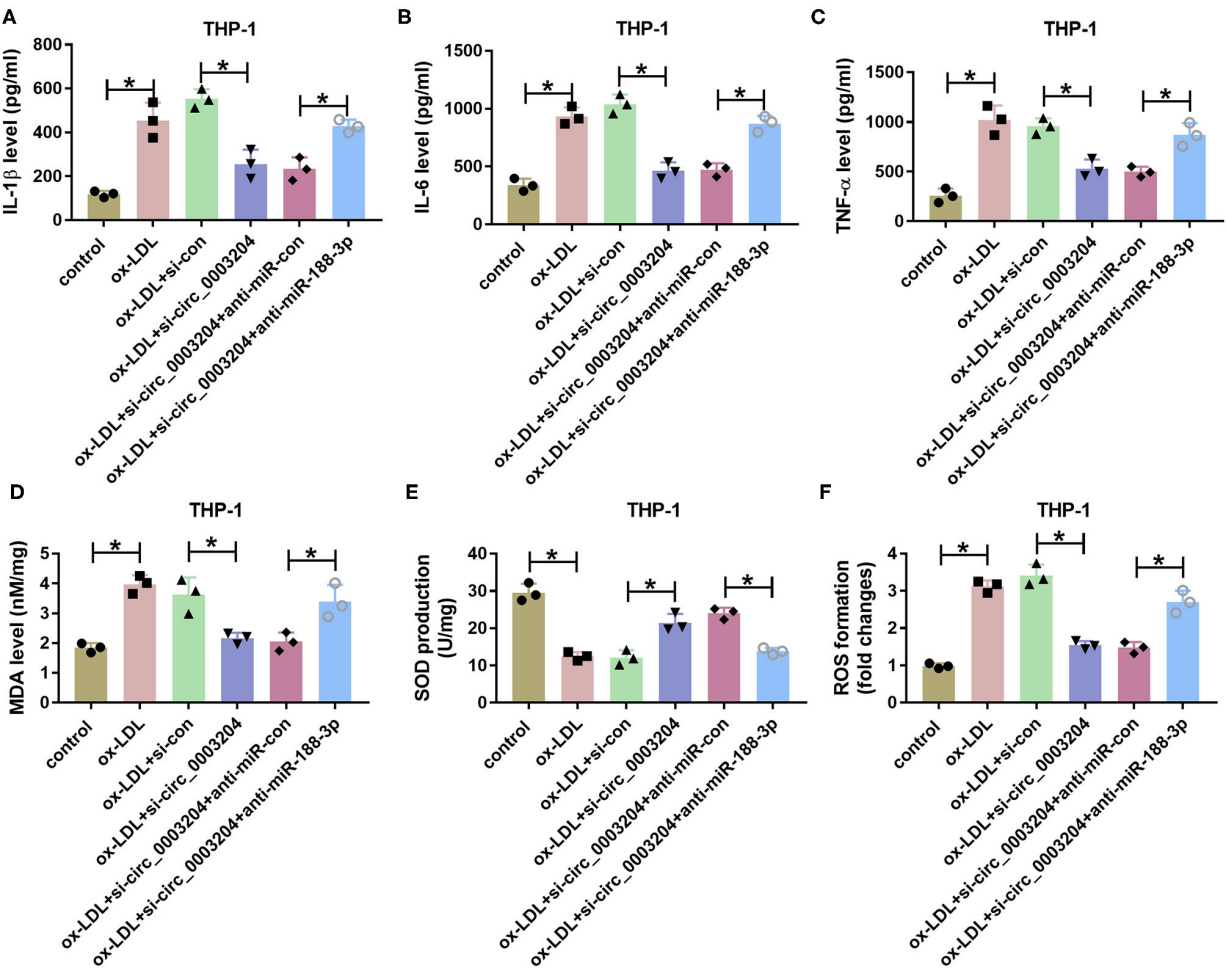
The effect of circ_0003204 and miR-188-3p on THP-1 cell inflammatory response and oxidative stress. The secretion levels of IL-1β, IL-6, and TNF-α **(A–C)** (one-way ANOVA), and levels of MDA, SOD and ROS **(D–F)** (one-way ANOVA) were measured in THP-1 cells transfected with si-con, si-circ_0003204, si-circ_0003204+anti-miR-con, or anti-miR-188-3p before exposure to ox-LDL. *p < 0.05. MDA, malondialdehyde; SOD, superoxide dismutase; ROS, reactive oxygen species.

In addition, the effect of circ_0003204 on ox-LDL-induced THP-1 cell damage was assessed. As described in Figures 5A–C, interference of circ_0003204 alleviated ox-LDL-caused inflammatory response via decreasing IL-1β, IL-6, and TNF-α, which was reversed via miR-188-3p knockdown. Besides, circ_0003204 silencing attenuated ox-LDL-induced oxidative stress via decreasing MDA and ROS levels and increasing SOD level, and these events were weakened by miR-188-3p knockdown (Figures 5D–F). These results suggested that circ_0003204 knockdown weakened ox-LDL-induced HCtAEC and THP-1 cell injury via mediating miR-188-3p.

### Transient Receptor Potential Canonical Channel 6 Knockdown Weakens Oxidized Low-Density Lipoprotein-Induced Injury in Human Carotid Artery Endothelial Cells and THP-1 Cells

To analyze the effects of TRPC6 knockdown on ox-LDL-induced injury in HCtAECs and THP-1 cells, we knocked out TRPC6 by transfection with si-TRPC6. The transfection efficiency is exhibited in Supplementary Figure 1A. Moreover, introduction of si-TRPC6 weakened the elevation of TRPC6 mediated by ox-LDL stimulation (Supplementary Figure 1B). As expected, TRPC6 downregulation weakened ox-LDL-induced viability and proliferation inhibition (Supplementary Figures 1C–E). Moreover, the elevated caspase-3 activity, apoptotic rate, and BAX protein levels and the decreased BCL-2 protein levels in HCtAECs and THP-1 cells caused by ox-LDL were mitigated by TRPC6 knockdown (Supplementary Figures 1F–I). In addition, ox-LDL-induced cell inflammation (Supplementary Figures 1K–M) and oxidative stress (Supplementary Figures 1N–P) were impaired after TRPC6 silencing. These results suggested that TRPC6 silencing weakens ox-LDL-induced injury in HCtAECs and THP-1 cells.

### MicroRNA-188-3p Overexpression Mitigates Oxidized Low-Density Lipoprotein-Induced Injury via Mediating Transient Receptor Potential Canonical Channel 6 in Human Carotid Artery Endothelial Cells and THP-1 Cells

**Figure 6:**
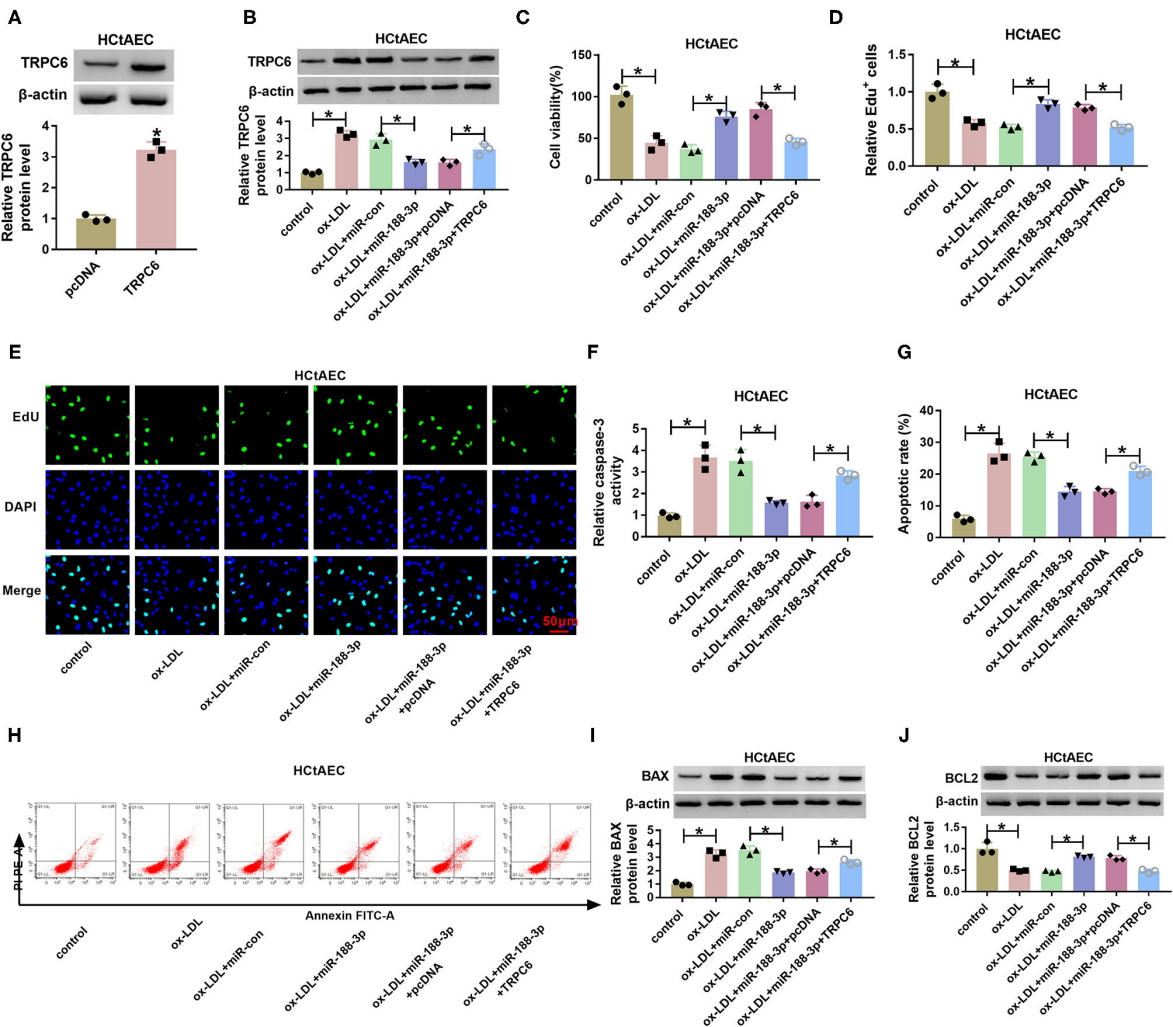
The effect of miR-188-3p and TRPC6 on HCtAEC viability and apoptosis. **(A)** TRPC6 protein level was measured (Student's *t*-test). **(B–J)** HCtAECs transfected with miR-con, miR-188-3p mimic, miR-188-3p mimic+pcDNA, or TRPC6 overexpression vector before stimulation of ox-LDL. **(B)** TRPC6 protein level was measured (one-way ANOVA). **(C–E)** Cell viability and cell proliferation were evaluated by MTT and EdU assays (one-way ANOVA). **(F)** Caspase-3 activity was examined (one-way ANOVA). **(G,H)** The apoptosis of HCtAECs was analyzed by flow cytometry analysis (one-way ANOVA). **(I,J)** The protein levels of BAX and BCL2 were measured (one-way ANOVA). *p < 0.05. HCtAEC, human carotid artery endothelial cell; TRPC6, transient receptor potential canonical channel 6; ox-LDL, oxidized low-density lipoprotein; EdU, 5-ethynyl-2′-deoxyuridine.

As exhibited in Figure 6A, transfection of the TRPC6 overexpression vector increased TRPC6 protein level in HCtAECs. To test whether miR-188-3p was associated with ox-LDL-induced HCtAEC injury through TRPC6, HCtAECs were transfected with miR-con, miR-188-3p mimic, miR-188-3p mimic+pcDNA, or TRPC6 overexpression vector before exposure to ox-LDL. As exhibited in Figures 6B–E, miR-188-3p overexpression mitigated ox-LDL-induced viability and proliferation suppression in HCtAECs, which was abrogated after TRPC6 overexpression. Furthermore, overexpression of miR-188-3p mitigated ox-LDL-caused apoptosis via reducing caspase-3 and modulating BAX and BCL2 protein levels, which was reversed by TRPC6 upregulation (Figures 6F–J).

**Figure 7:**
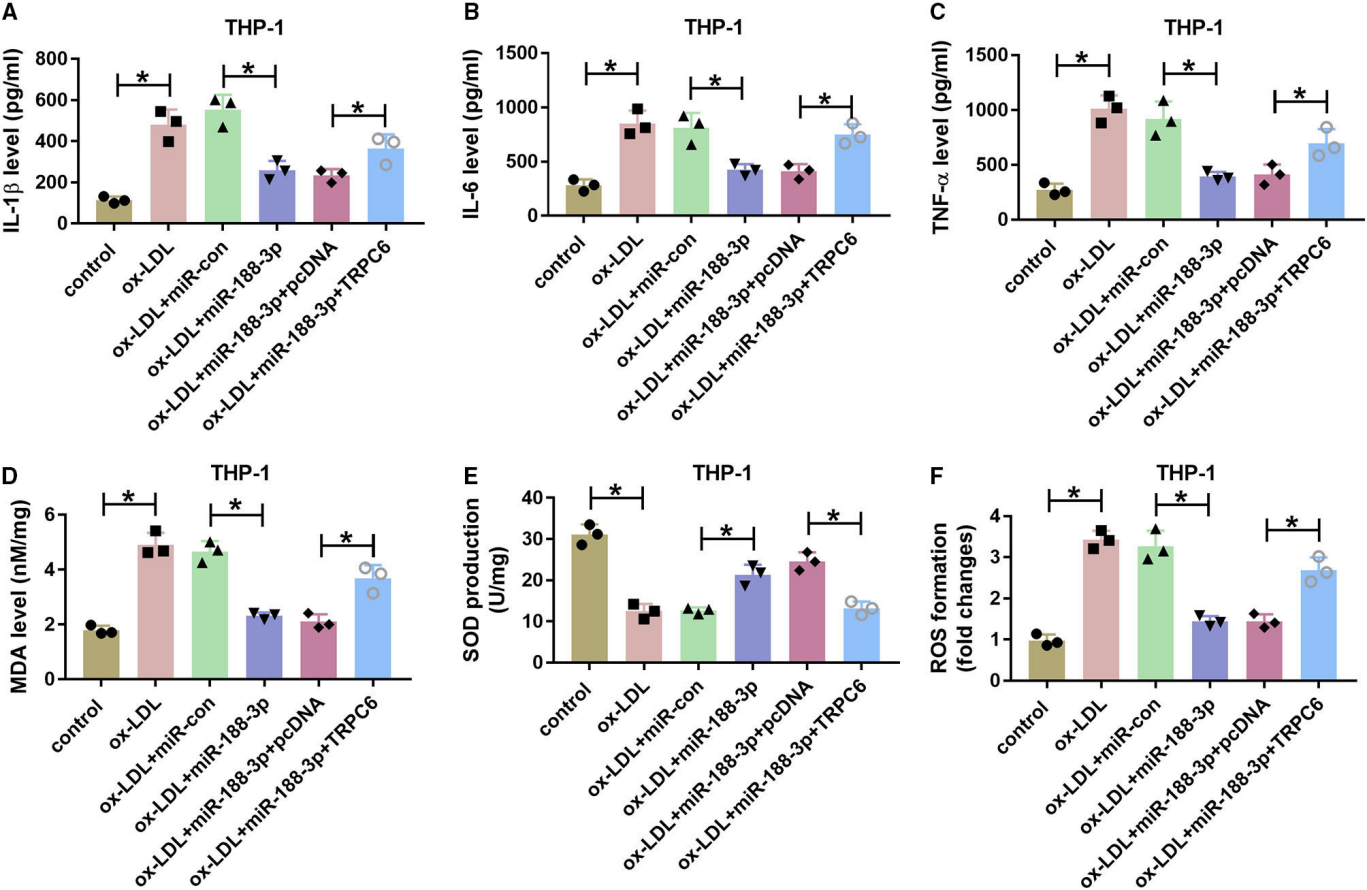
The effect of miR-188-3p and TRPC6 on THP-1 cell inflammatory response and oxidative stress. The secretion levels of IL-1β, IL-6, and TNF-α **(A–C)** (one-way ANOVA) and levels of MDA, SOD and ROS **(D–F)** (one-way ANOVA) were examined in THP-1 cells transfected with miR-con, miR-188-3p mimic, miR-188-3p mimic+pcDNA, or TRPC6 overexpression vector before exposure to ox-LDL. *p < 0.05. TRPC6, transient receptor potential canonical channel 6; MDA, malondialdehyde; SOD, superoxide dismutase; ROS, reactive oxygen species; ox-LDL, oxidized low-density lipoprotein.

Additionally, the effect of miR-188-3p on ox-LDL-induced damage in THP-1 cells was tested. As shown in Figures 7A–C, overexpression of miR-188-3p alleviated ox-LDL-induced inflammatory response via reducing the levels of IL-1β, IL-6, and TNF-α in THP-1 cells, which were overturned by TRPC6 overexpression. Besides, miR-188-3p overexpression attenuated ox-LDL-induced oxidative stress by inhibiting MDA and ROS levels and elevating SOD level, while these events were reversed after TRPC6 restoration (Figures 7D–F). These data indicated that miR-188-3p overexpression attenuated ox-LDL-induced damage in HCtAECs and THP-1 cells via targeting TRPC6.

## Discussion

AS causes high morbidity and mortality all around the world (18). Carotid artery AS is a common form of AS (19). The inflammatory response, oxidative stress, and endothelial cell apoptosis are implicated in AS progression (20–22). CircRNAs play a key role in the development of AS (23). This research focused on the function and mechanism of circ_0003204 in the regulation of ox-LDL-induced inflammatory response, oxidative stress, and endothelial cell apoptosis. Here, we first found that circ_0003204 knockdown could mitigate ox-LDL-induced injury in HCtAECs and THP-1 cells.

We established ox-LDL-stimulated HCtAECs and THP-1 cells, and we found that circ_0003204 expression was elevated in the two cell lines, which was consistent with that in HAECs or HUVECs (8, 9). Hence, we assumed the increase in circ_0003204 induced by abnormal ox-LDL might be correlated with carotid artery AS development. Previous studies suggested that ox-LDL could induce endothelial cell injury and that ox-LDL-stimulated HCtAECs could be used to assess the pathogenesis of carotid artery AS *in vitro* (15, 24, 25). Similarly, our study also found that ox-LDL caused HCtAEC viability inhibition and apoptosis promotion and that circ_0003204 knockdown weakened ox-LDL-exposed HCtAEC injury. In addition, ox-LDL-mediated THP-1 cell damage is also involved in the development of carotid artery AS (15, 26, 27). By detecting the pro-inflammatory cytokine levels and oxidative stress-related markers, we confirmed that circ_0003204 silencing attenuated ox-LDL-induced inflammatory response and oxidative stress in THP-1 cells. Collectively, inhibition of circ_0003204 played an inhibiting effect in ox-LDL-induced injury in AS.

Previous studies suggested that circRNA-mediated ceRNA network is the important mechanism in cardiovascular diseases and ox-LDL-induced injury (28, 29). The former work has confirmed that circ_0003204 could act as a ceRNA to regulate the miR-370-3p/TGFβR2 axis (8). Here, we first explored the potential association among circ_0003204, miR-188-3p, and TRPC6. In addition, we found that circ_0003204 could positively regulate TRPC6 expression by binding to miR-188-3p, indicating that circ_0003204 might function as a ceRNA for miR-188-3p to mediate TRPC6. Our study found that miR-188-3p overexpression weakened ox-LDL-induced viability inhibition and apoptosis promotion in HCtAECs. Furthermore, we confirmed miR-188-3p weakened ox-LDL-induced inflammatory response in THP-1 cells, which was similar to that in a previous study (12). Besides, our results also displayed that miR-188-3p could mitigate ox-LDL-induced oxidative stress in THP-1 cells. These data indicated the protective function of miR-188-3p in ox-LDL-induced damage. Additionally, miR-188-3p knockdown reversed the effect of circ_0003204 silencing on ox-LDL-induced damage in the two cell lines, implying that circ_0003204 could regulate ox-LDL-induced cell injury by mediating miR-188-3p in HCtAECs and THP-1 cells.

Next, we validated the targeting interaction between miR-188-3p and TRPC6. Negri et al. and Thilo et al. reported that TRPC6 was abnormally expressed in vascular endothelial cells, and its elevation was associated with cardiovascular disease development (30, 31). Moreover, Zhang et al. suggested that TRPC6 could contribute to AS development by promoting endothelial cell apoptosis (14). Similarly, our study also confirmed that the inhibitive role of miR-188-3p on ox-LDL-mediated cell injury in HCtAECs and THP-1 cells was partly reversed by TRPC6 overexpression. Collectively, we concluded that circ_0003204 could regulate ox-LDL-induced cell injury by mediating miR-188-3p and TRPC6. However, it did not indicate the biological role of circ_0003204 in carotid artery AS *in vivo* because of the alteration of microenvironment. The animal models have been widely used to assess the pathogenesis of AS *in vivo* (32). Therefore, the preclinical experiments using animals would be performed to test the function and mechanism of circ_0003204 in carotid artery AS in future.

**Figure 8:**
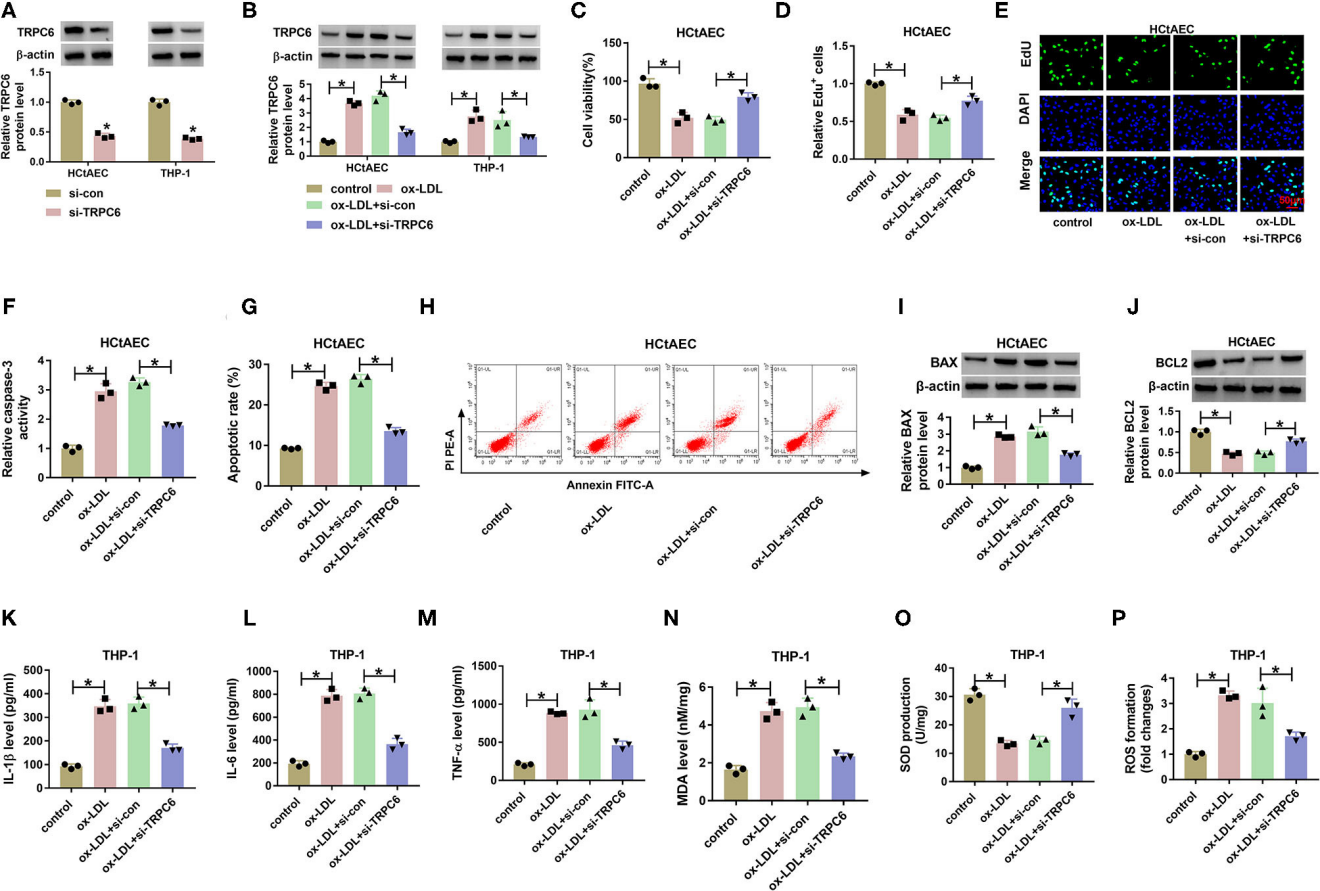
**(A–P)** Circ_0003204 regulated ox-LDL-induced injury in HCtAECs and THP-1 cells via miR-188-3p/TRPC6 axis. ox-LDL, oxidized low-density lipoprotein; HCtAECs, human carotid artery endothelial cells; TRPC6, transient receptor potential canonical channel 6.

In conclusion, circ_0003204 knockdown could attenuate ox-LDL-induced HCtAEC apoptosis and THP-1 cell inflammatory response and oxidative stress via modulating miR-188-3p/TRPC6 axis in a ceRNA network (Figure 8). This study indicated that this ceRNA crosstalk might be associated with ox-LDL-induced injury in carotid artery AS and that circ_0003204 might act as a target for carotid artery AS therapy.

## Data Availability Statement

The original contributions presented in the study are included in the article/Supplementary Material, further inquiries can be directed to the corresponding author/s.

## Author Contributions

WP designed and performed the research and wrote the manuscript. SL, SC, JY, and ZS analyzed the data. All authors have read and approved the final manuscript.

## Funding

This work was supported by the Natural Science Foundation of China (No. 31600755).

## Conflict of Interest

The authors declare that the research was conducted in the absence of any commercial or financial relationships that could be construed as a potential conflict of interest.

## Publisher's Note

All claims expressed in this article are solely those of the authors and do not necessarily represent those of their affiliated organizations, or those of the publisher, the editors and the reviewers. Any product that may be evaluated in this article, or claim that may be made by its manufacturer, is not guaranteed or endorsed by the publisher.

## Supplementary Material

Supplementary Figure 1 TRPC6 knockdown weakens ox-LDL-induced injury in HCtAEC and THP-1 cells. **(A)** Western blot showing TRPC6 protein levels in in HCtAEC and THP-1 cells transfected with si-con or si-TRPC6 (Student's *t*-test). **(B–P)** HCtAEC cells were transfected with si-con or si-TRPC6 and then treated with ox-LDL. **(B)** Western blot showing TRPC6 protein levels in HCtAEC and THP-1 cells (one-way ANOVA). **(C–E)** MTT and EdU assays (one-way ANOVA) were performed to analyze cell viability and proliferation (one-way ANOVA). **(F)** Evaluation of Caspase-3 activity was performed (one-way ANOVA). (**G,H**) Flow cytometry assays were carried out to analyze cell apoptosis (one-way ANOVA). (**I,J**) The protein levels of BAX and BCL2 in HCtAEC cells were measured (one-way ANOVA). **(K–P)** Measurements of IL-1β, IL-6,TNF-α, MDA, SOD, and ROS were conducted (one-way ANOVA). ^*^*P* < 0.05.
