# Supplementary material for: Hsa_circ_0003204 Knockdown Weakens Ox-LDL-Induced Cell Injury by Regulating miR-188-3p/TRPC6 Axis in Human Carotid Artery Endothelial Cells and THP-1 Cells
Source: Front Cardiovasc Med. 2021 Nov 29;8:731890. doi: 10.3389/fcvm.2021.731890 (PMC8666549; doi:10.3389/fcvm.2021.731890)
Supplement: Supplementary Figure 1 — TRPC6 knockdown weakens ox-LDL-induced injury in HCtAEC and THP-1 cells. (A) Western blot showing TRPC6 protein levels in in HCtAEC and THP-1 cells transfected with si-con or si-TRPC6 (Student's t-test). (B–P) HCtAEC cells were transfected with si-con or si-TRPC6 and then treated with ox-LDL. (B) Western blot showing TRPC6 protein levels in HCtAEC and THP-1 cells (one-way ANOVA). (C–E) MTT and EdU assays (one-way ANOVA) were performed to analyze cell viability and proliferation (one-way ANOVA). (F) Evaluation of Caspase-3 activity was performed (one-way ANOVA). (G,H) Flow cytometry assays were carried out to analyze cell apoptosis (one-way ANOVA). (I,J) The protein levels of BAX and BCL2 in HCtAEC cells were measured (one-way ANOVA). (K–P) Measurements of IL-1β, IL-6,TNF-α, MDA, SOD, and ROS were conducted (one-way ANOVA). *P < 0.05. [file Image_1.PDF]

1E-β-actin

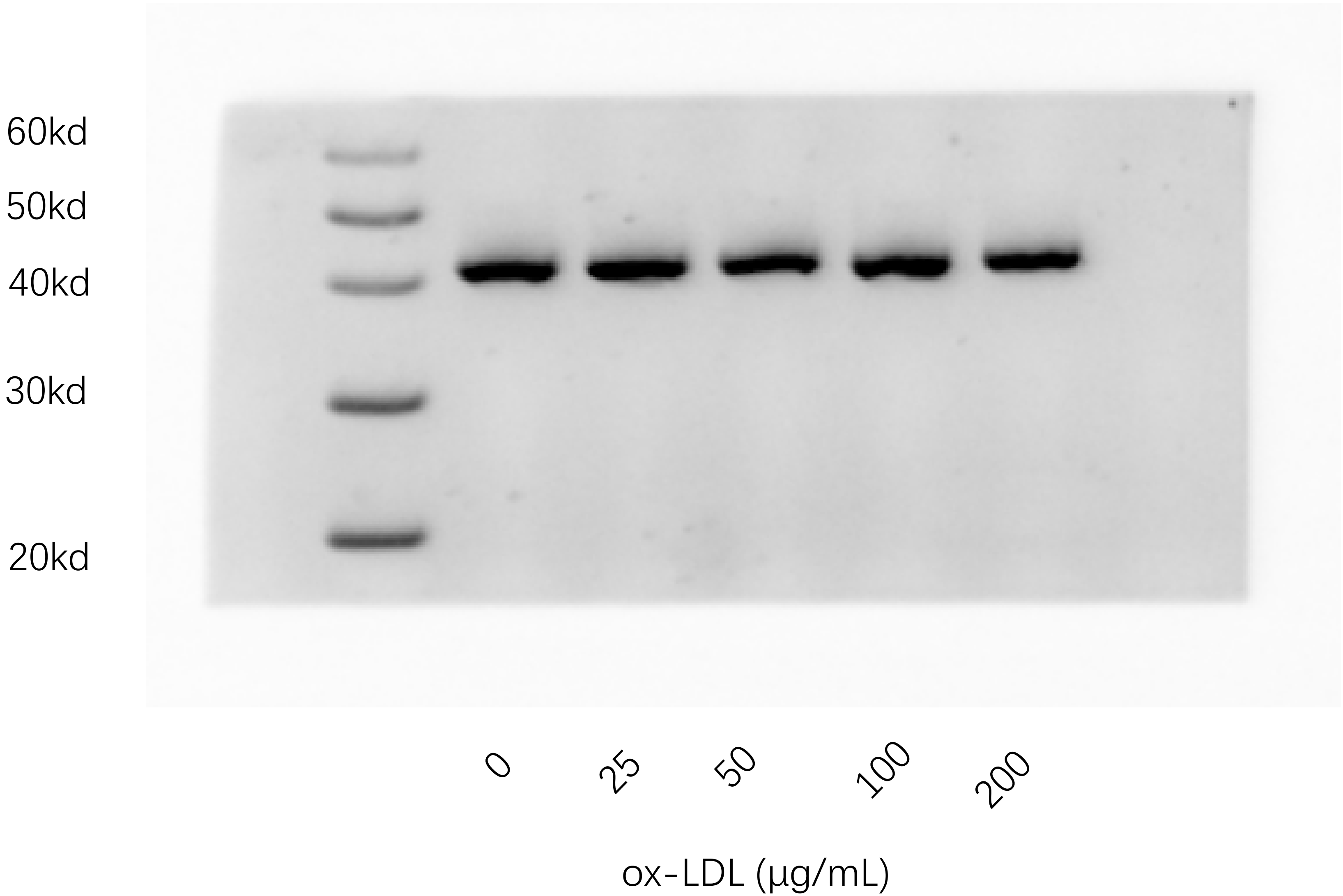

1E-TRPC6

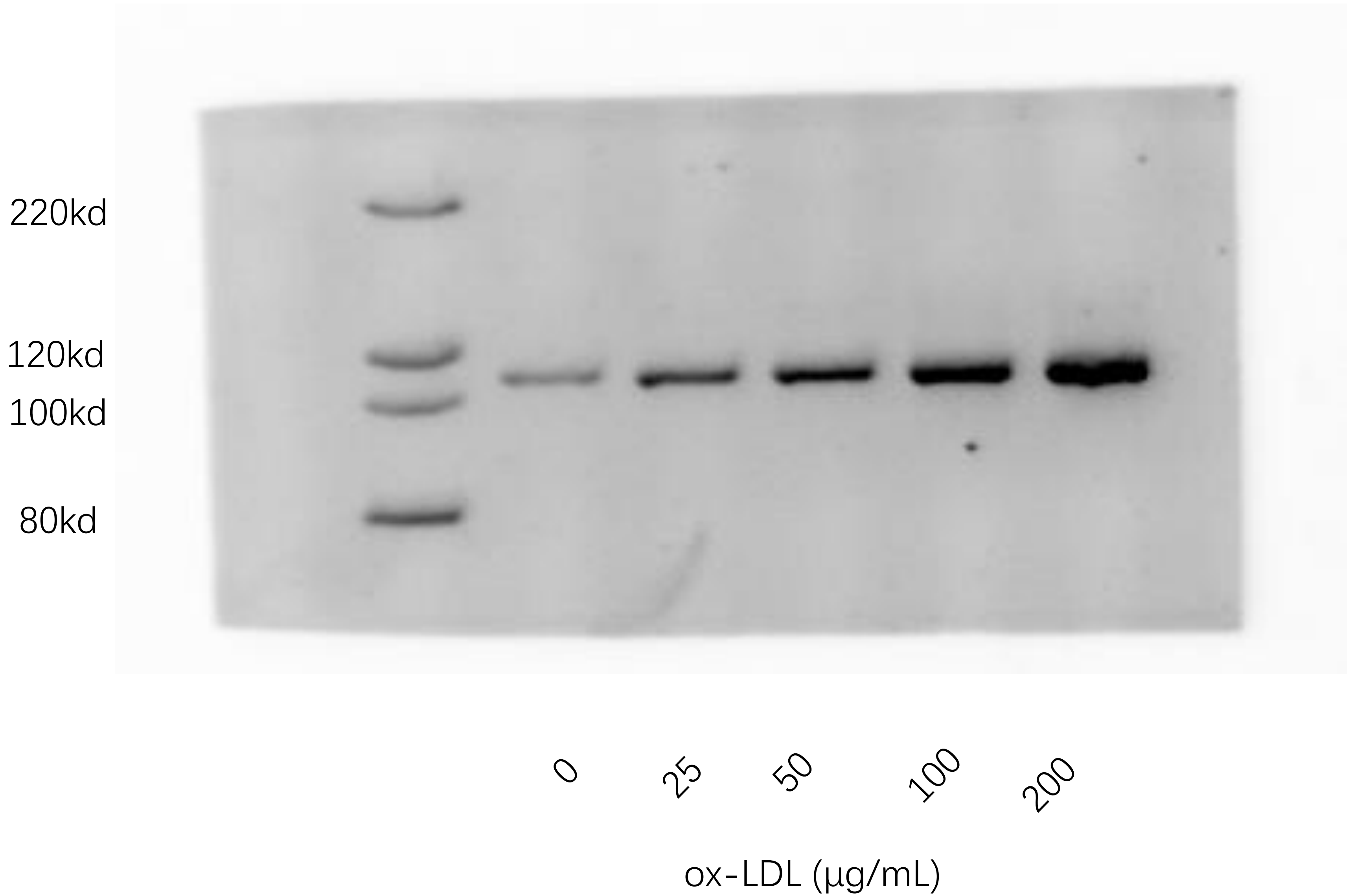

1F-β-actin

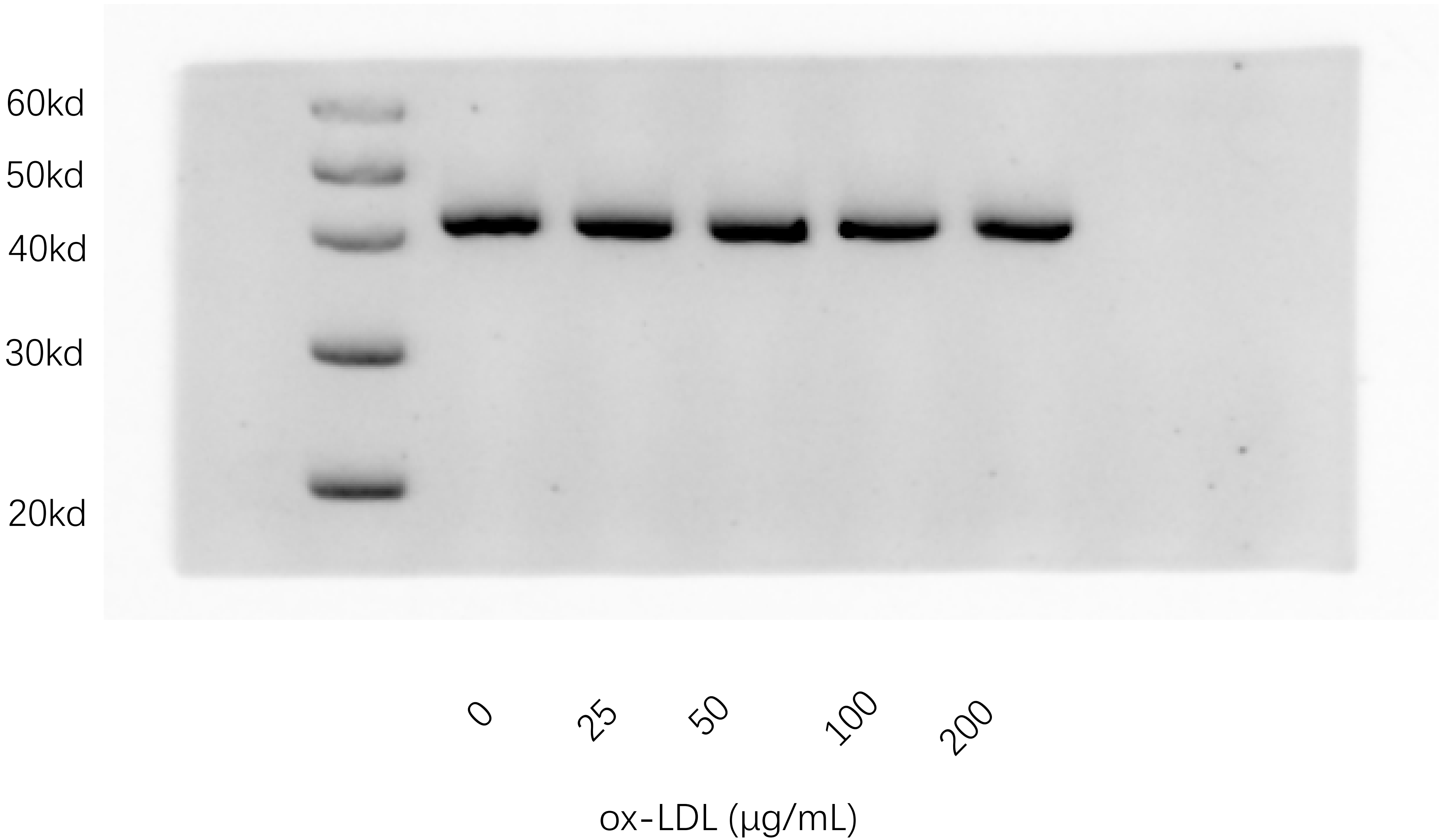

1F-TRPC6

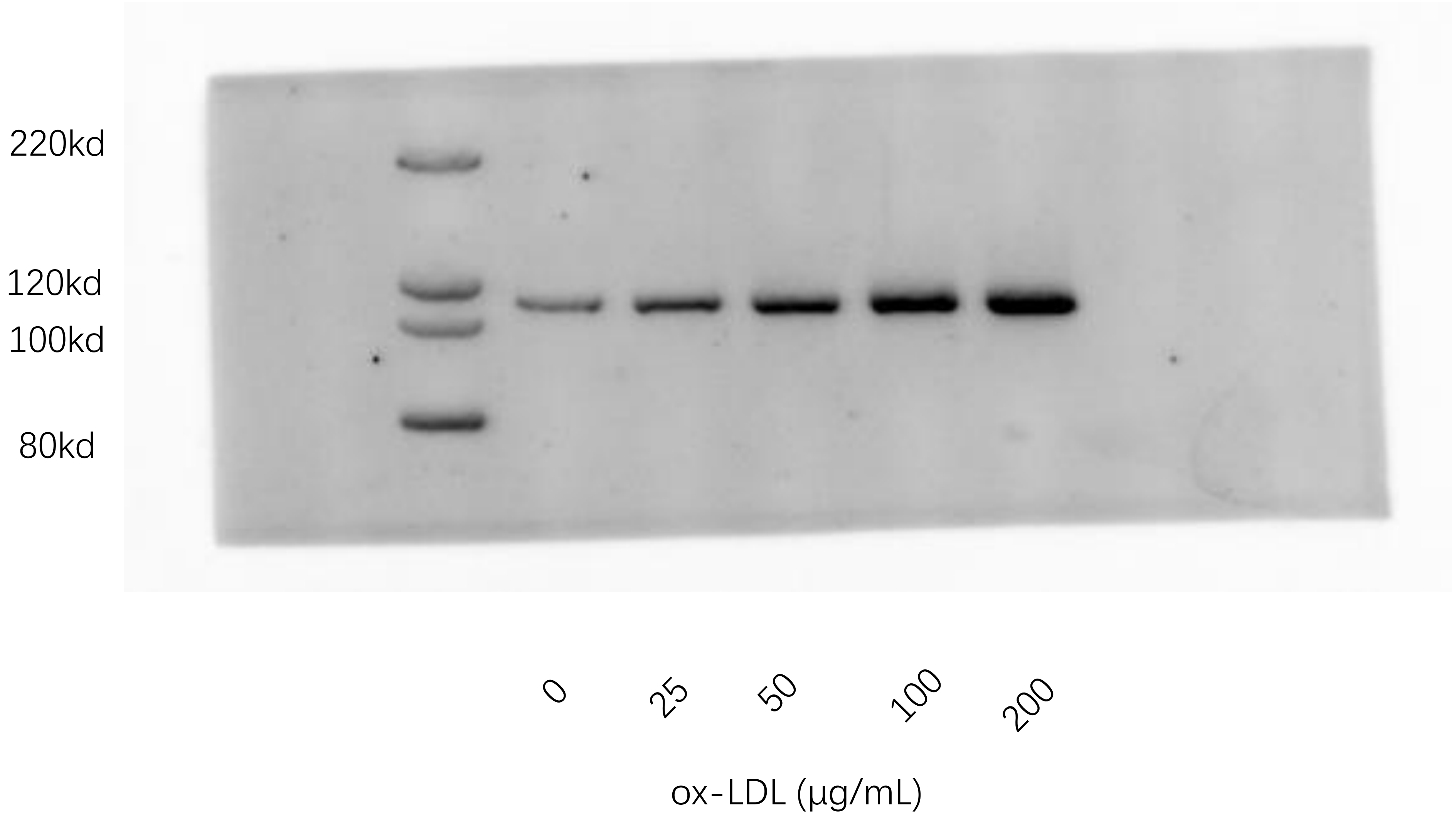

3G-β-actin

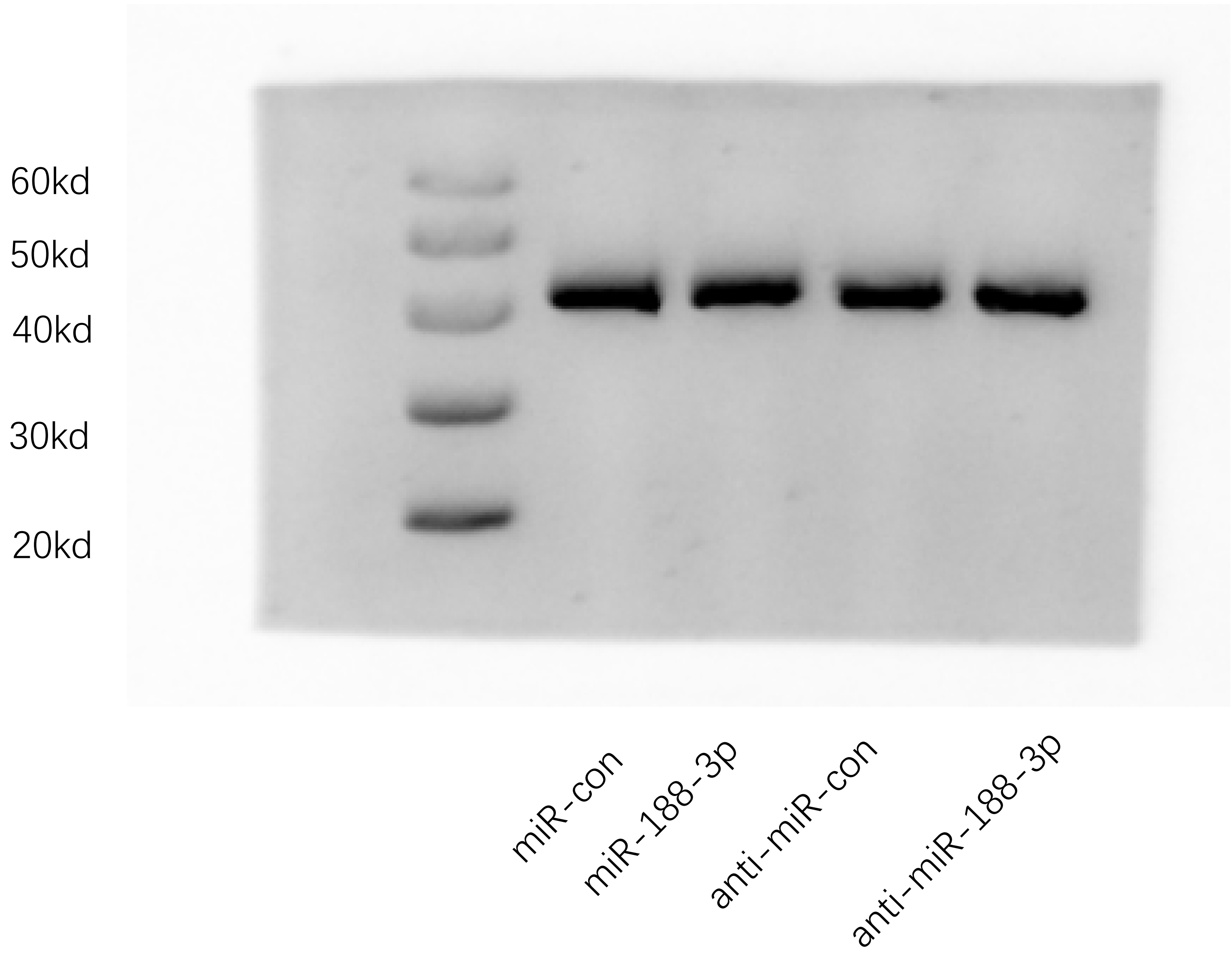
60kd
50kd
40kd
30kd
20kd
miR-con
miR-188-3p
anti-miR-con
anti-miR-188-3p

3G-TRPC6

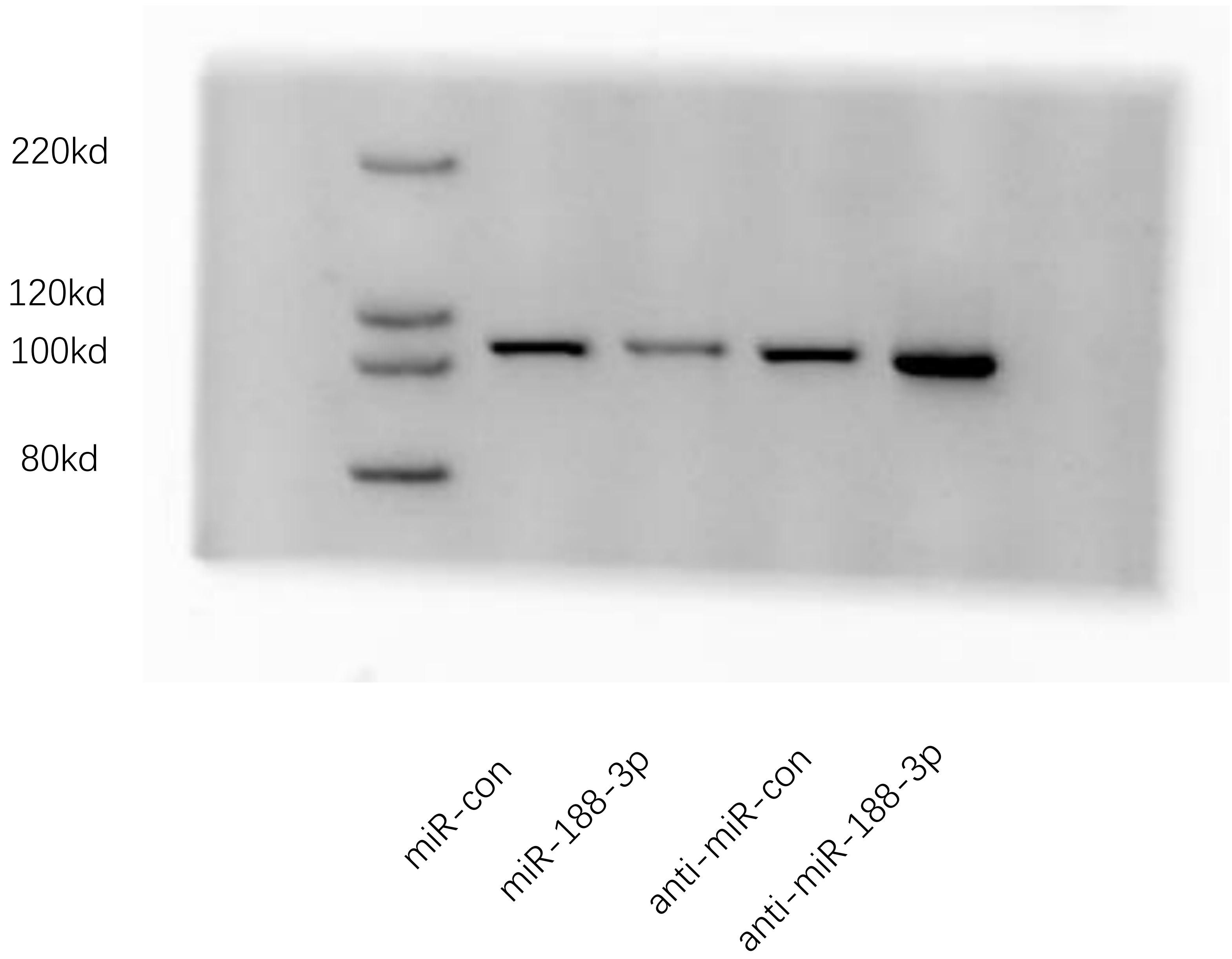
220kd
120kd
100kd
80kd
miR-con
miR-188-3p
anti-miR-con
anti-miR-188-3p

3H-β-actin

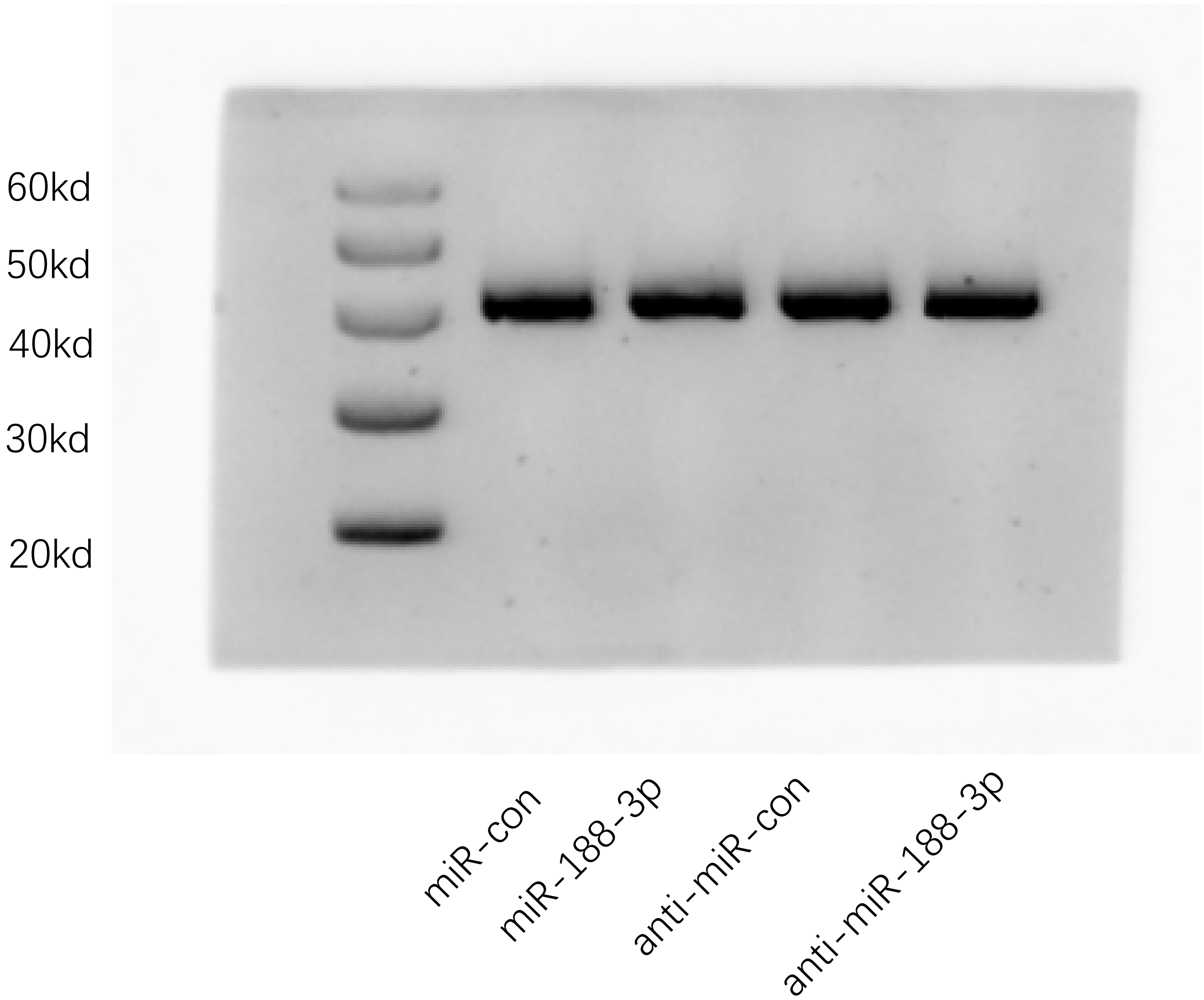
60kd
50kd
40kd
30kd
20kd
miR-con
miR-188-3p
anti-miR-con
anti-miR-188-3p

3H-TRPC6

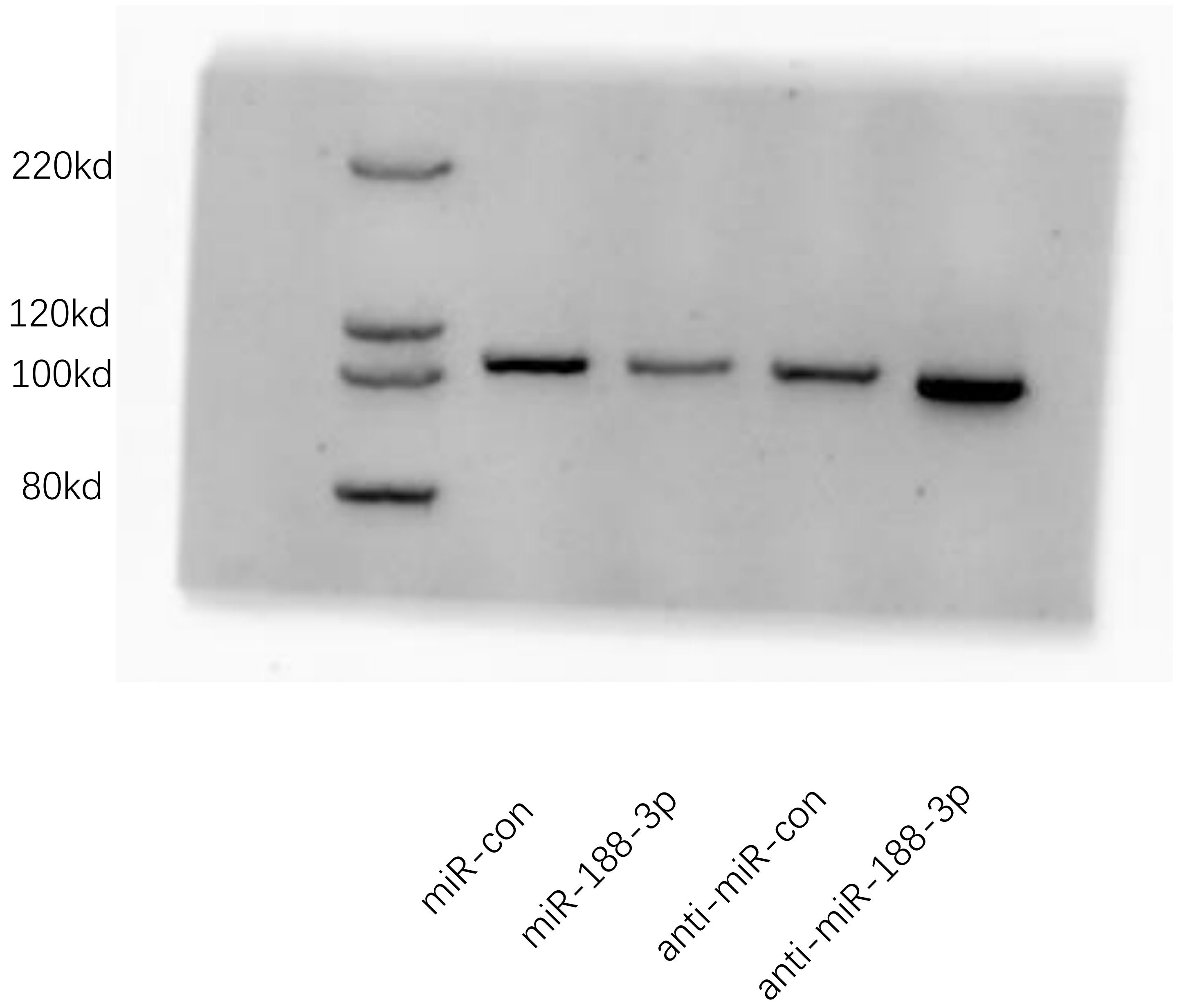
220kd
120kd
100kd
80kd
miR-con
miR-188-3p
anti-miR-con
anti-miR-188-3p

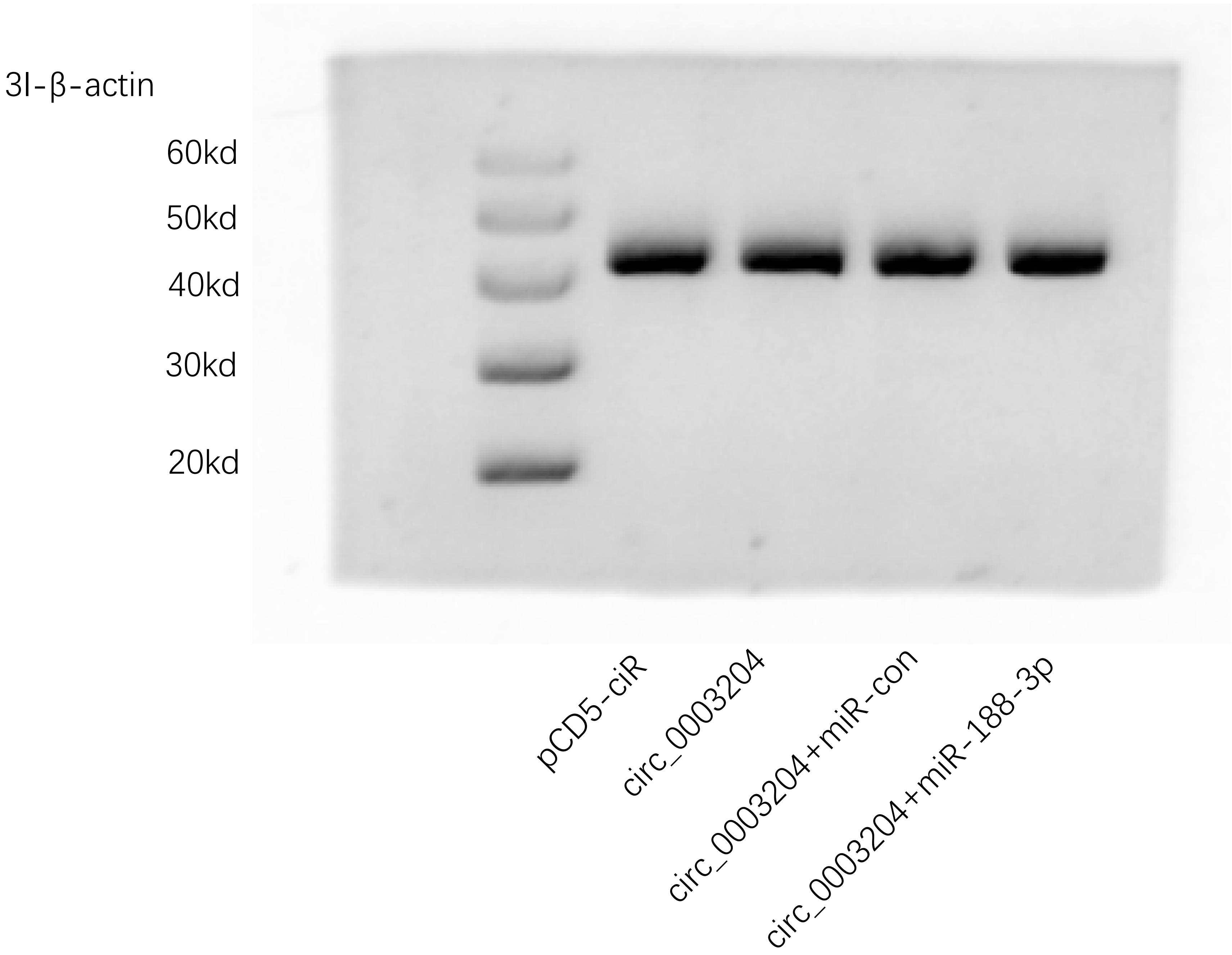
3I-β-actin
60kd
50kd
40kd
30kd
20kd
pCD5-ciR
circ_0003204
circ_0003204+miR-con
circ_0003204+miR-188-3p

3I-TRPC6

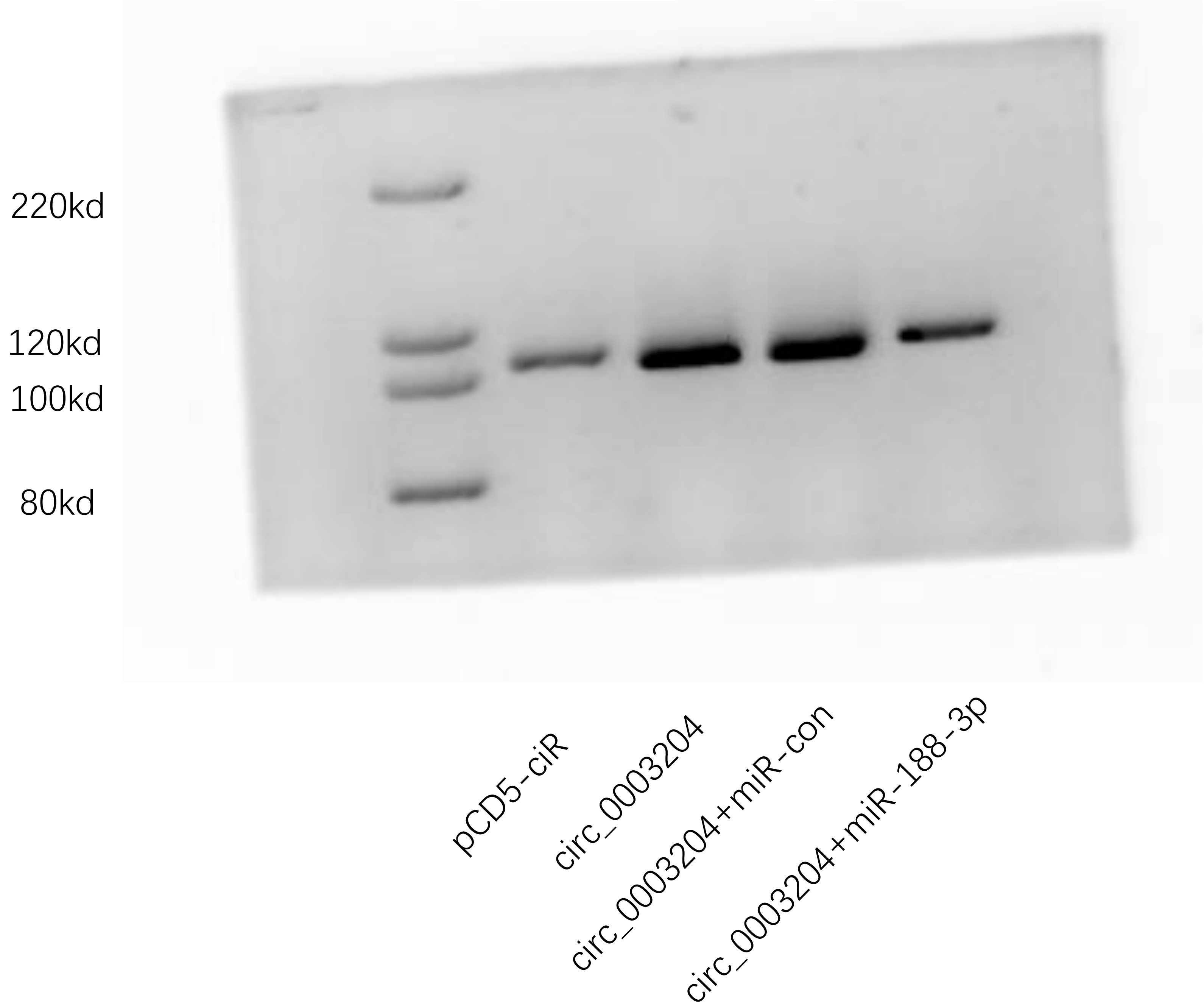
220kd
120kd
100kd
80kd
pCD5-ciR
circ_0003204
circ_0003204+miR-con
circ_0003204+miR-188-3p

3J-β-actin

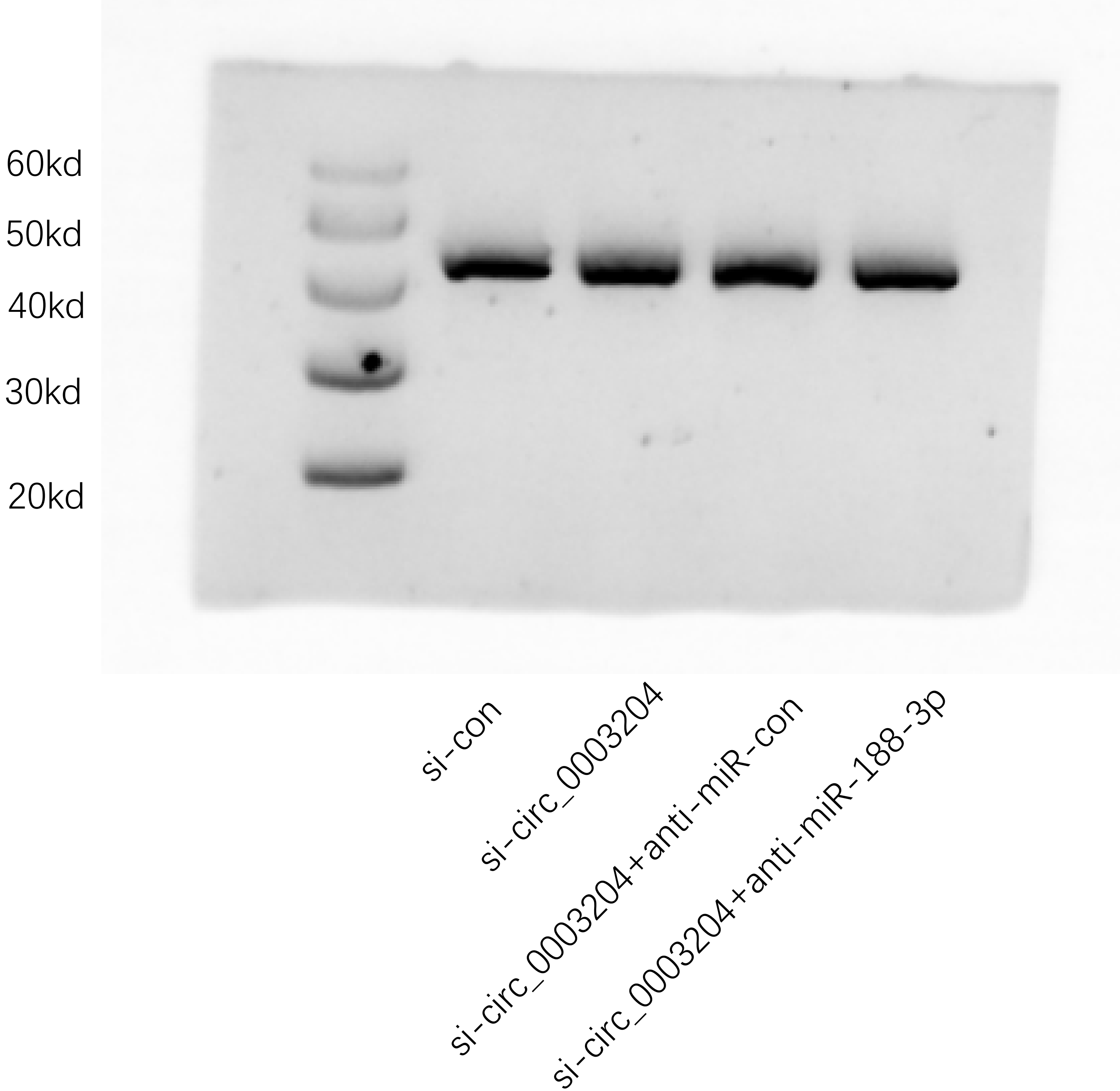
60kd
50kd
40kd
30kd
20kd
si-con
si-circ_0003204
si-circ_0003204+anti-miR-con
si-circ_0003204+anti-miR-188-3p

3J-TRPC6

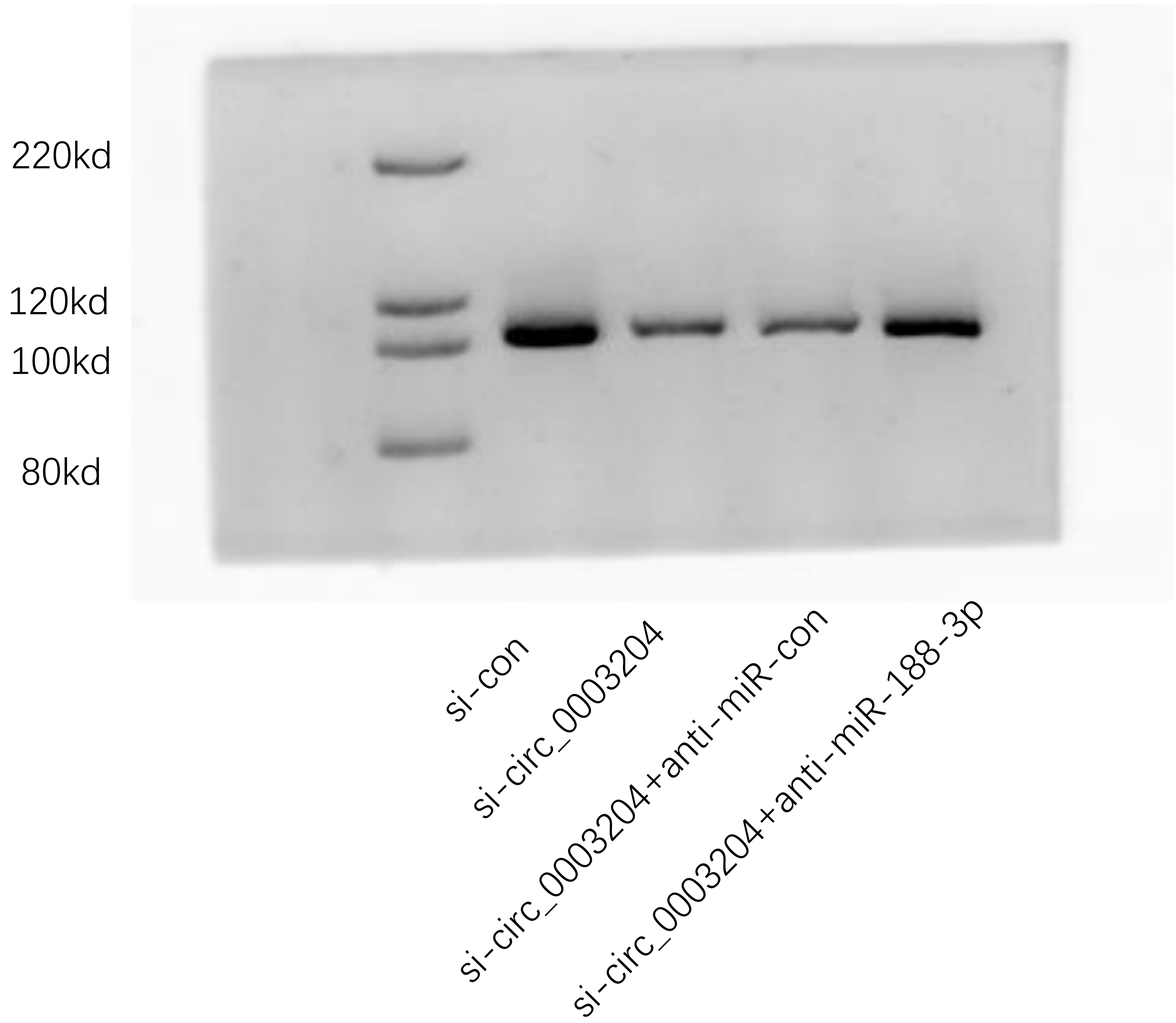
220kd
120kd
100kd
80kd
si-con
si-circ_0003204
si-circ_0003204+anti-miR-con
si-circ_0003204+anti-miR-188-3p

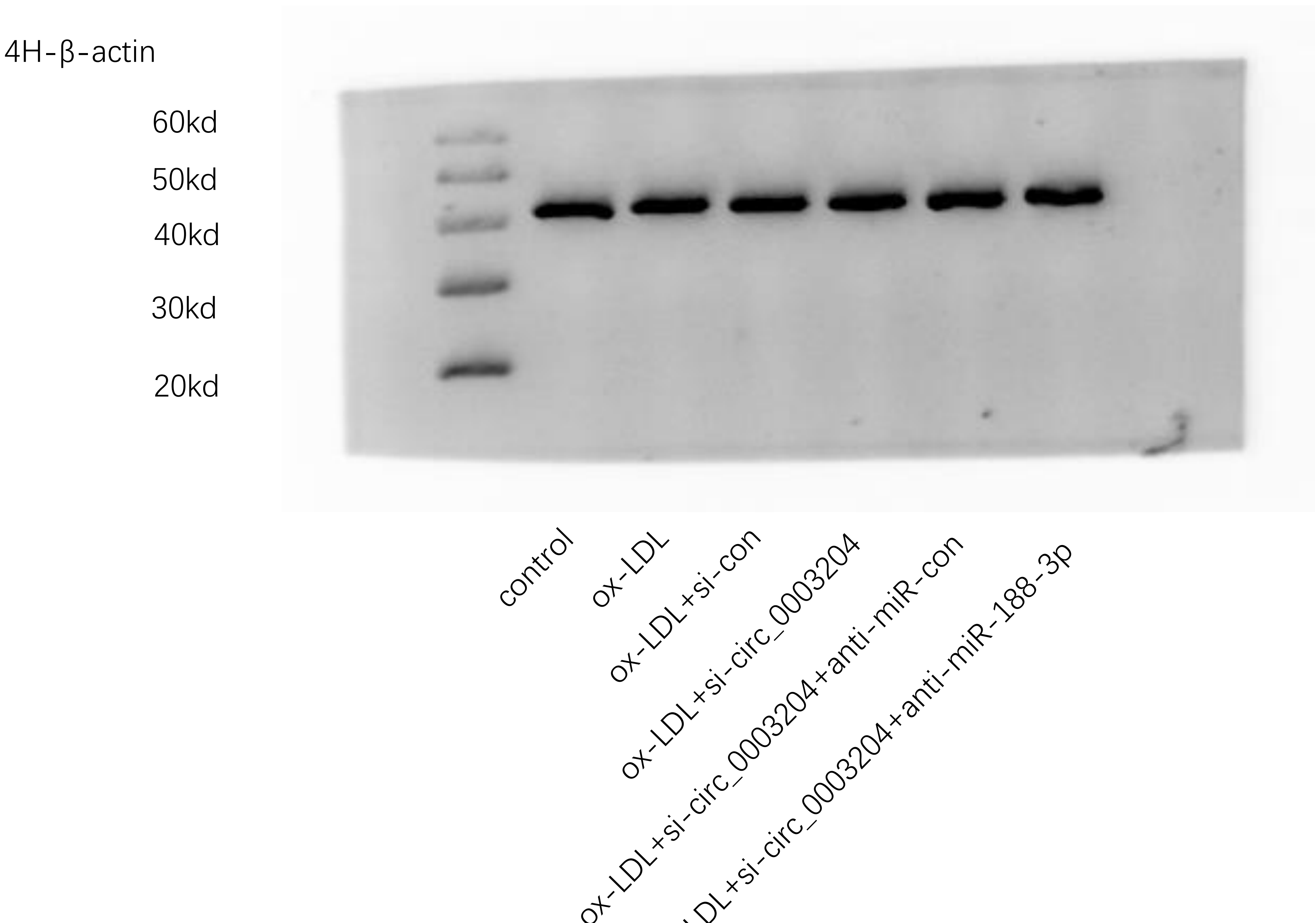
4H-β-actin
60kd
50kd
40kd
30kd
20kd
control
ox-LDL
ox-LDL+si-con
ox-LDL+si-circ_0003204
ox-LDL+si-circ_0003204+anti-miR-con
LDL+si-circ_0003204+anti-miR-188-3p

4H-BAX

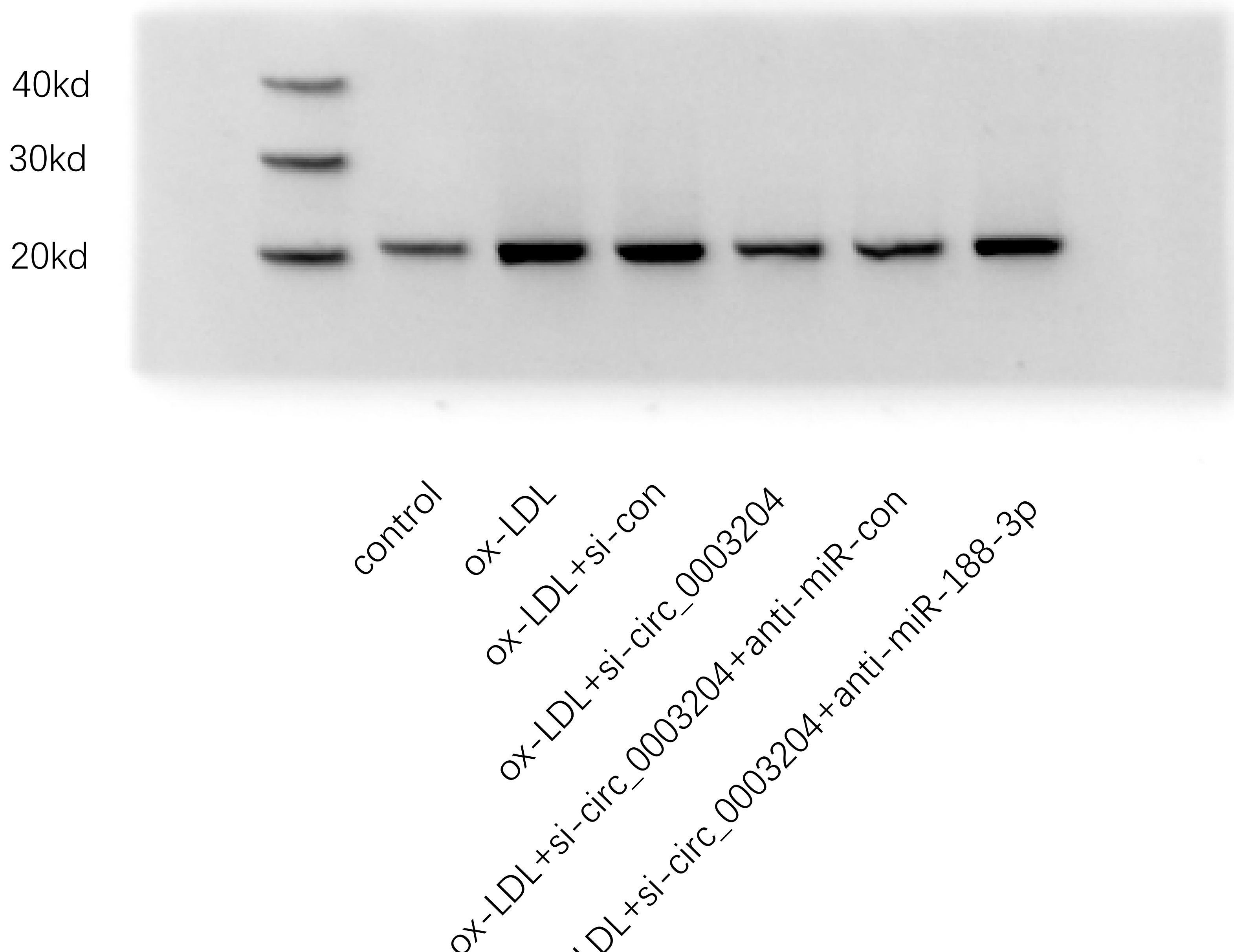
40kd
30kd
20kd
control
ox-LDL
ox-LDL+si-con
ox-LDL+si-circ_0003204
ox-LDL+si-circ_0003204+anti-miR-con
LDL+si-circ_0003204+anti-miR-188-3p

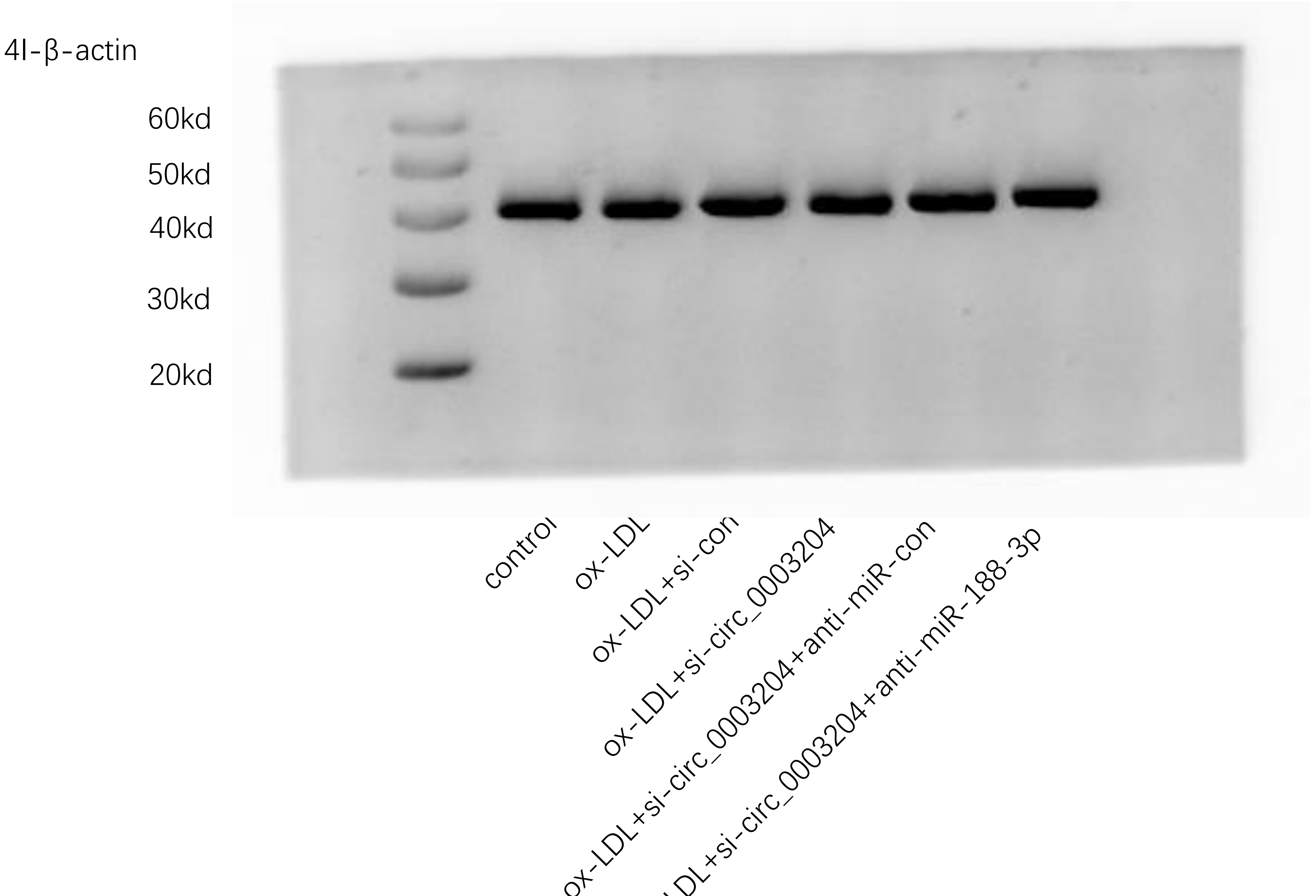
4I-β-actin
60kd
50kd
40kd
30kd
20kd
control
ox-LDL
ox-LDL+si-con
ox-LDL+si-circ_0003204
ox-LDL+si-circ_0003204+anti-miR-con
LDL+si-circ_0003204+anti-miR-188-3p

4-BCL2

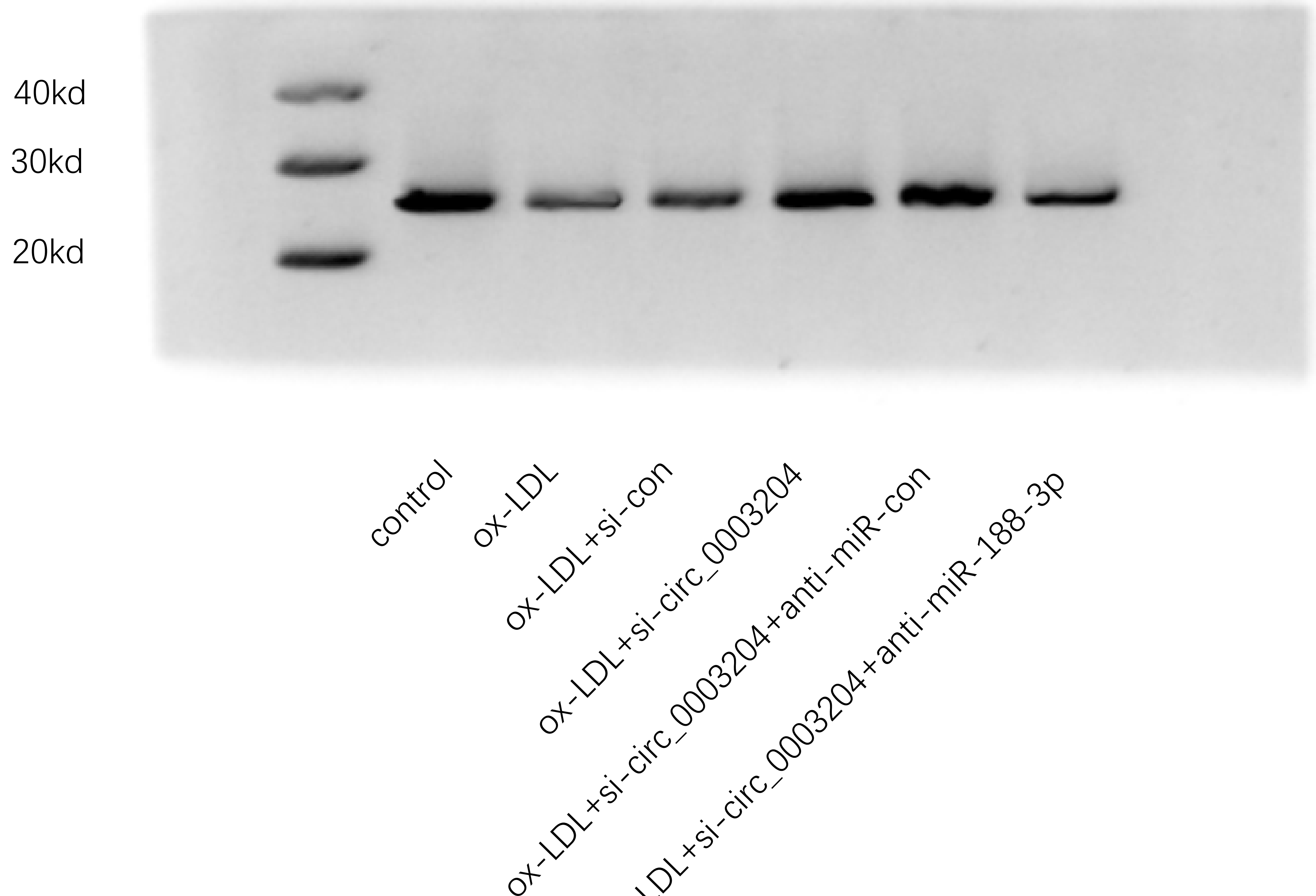
40kd
30kd
20kd
control
ox-LDL
ox-LDL+si-con
ox-LDL+si-circ_0003204
ox-LDL+si-circ_0003204+anti-miR-con
LDL+si-circ_0003204+anti-miR-188-3p

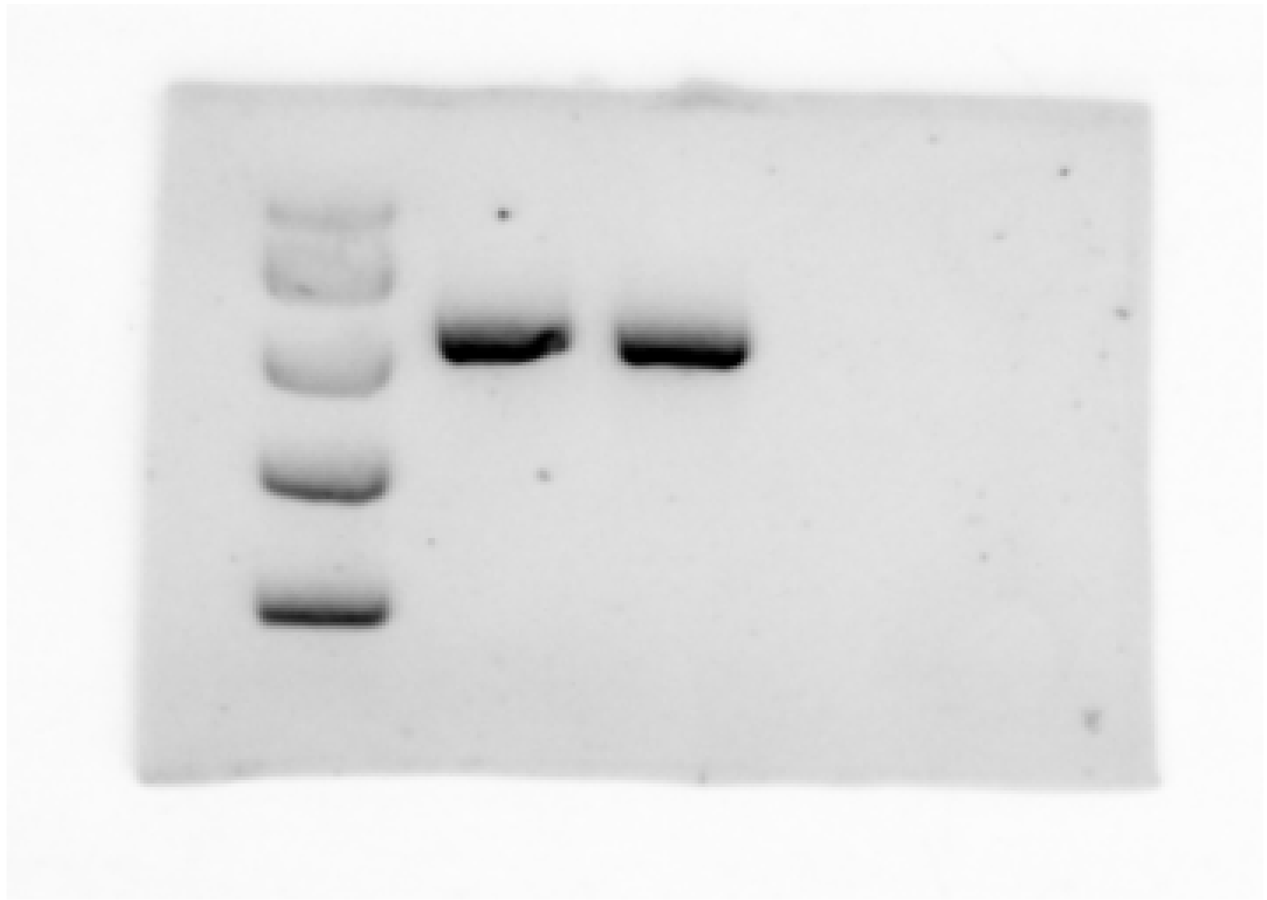
6A-β-actin
60kd
50kd
40kd
30kd
20kd
pcDNA
TRPC6

6A-TRPC6

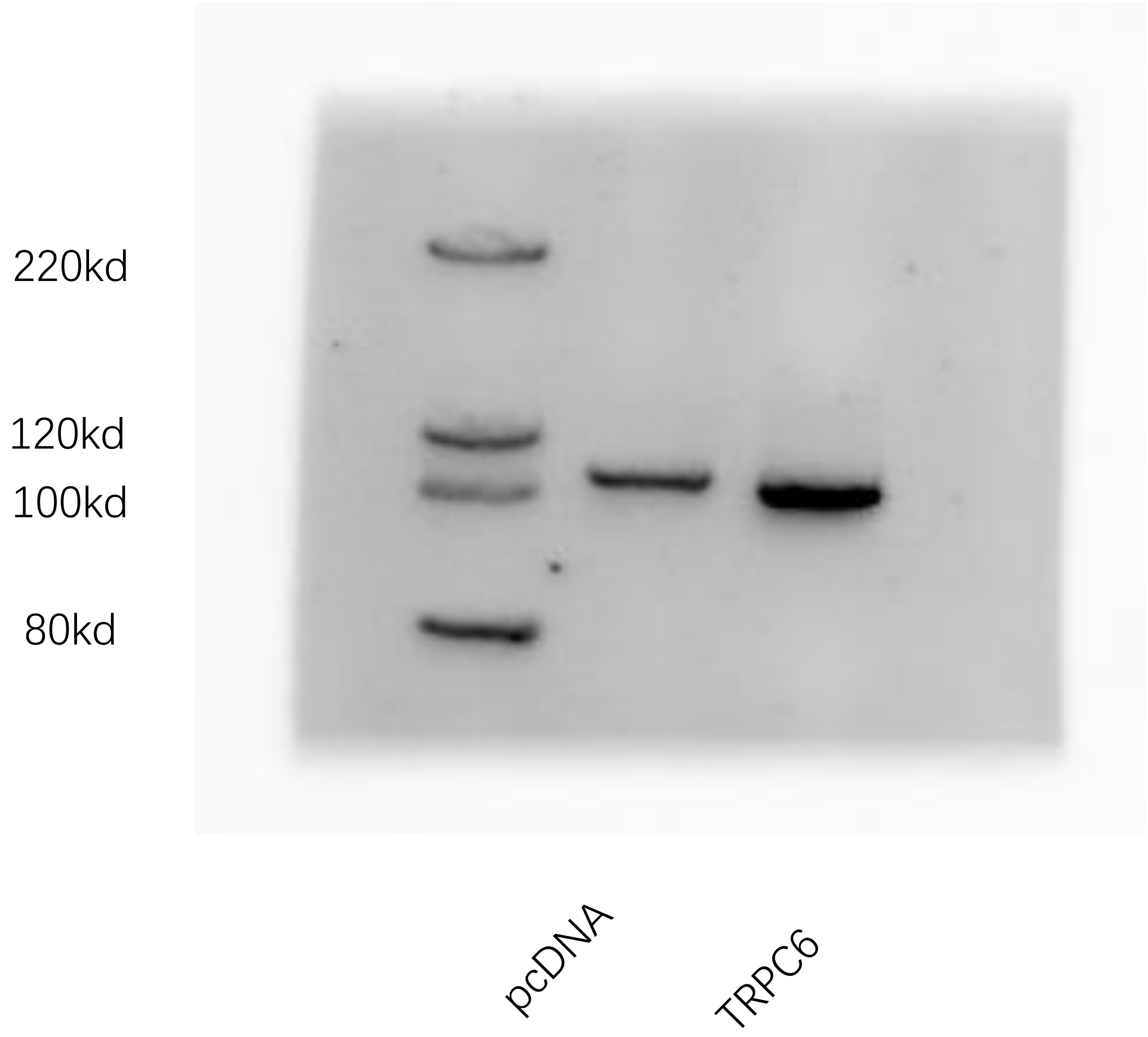

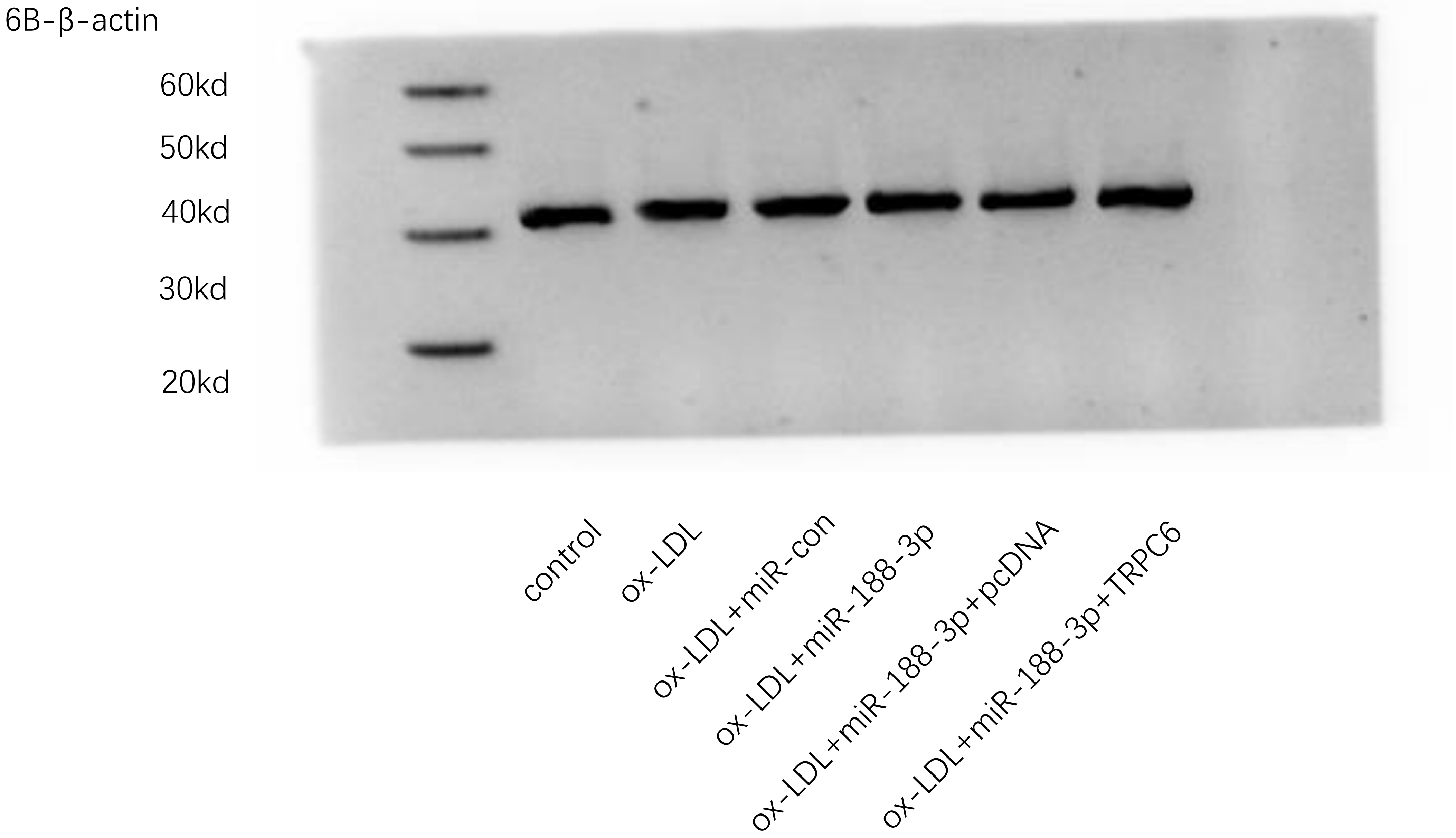

6B-β-actin
60kd
50kd
40kd
30kd
20kd
control
ox-LDL
ox-LDL+miR-con
ox-LDL+miR-188-3p
ox-LDL+miR-188-3p+pcDNA
ox-LDL+miR-188-3p+TRPC6

6B-TRPC6

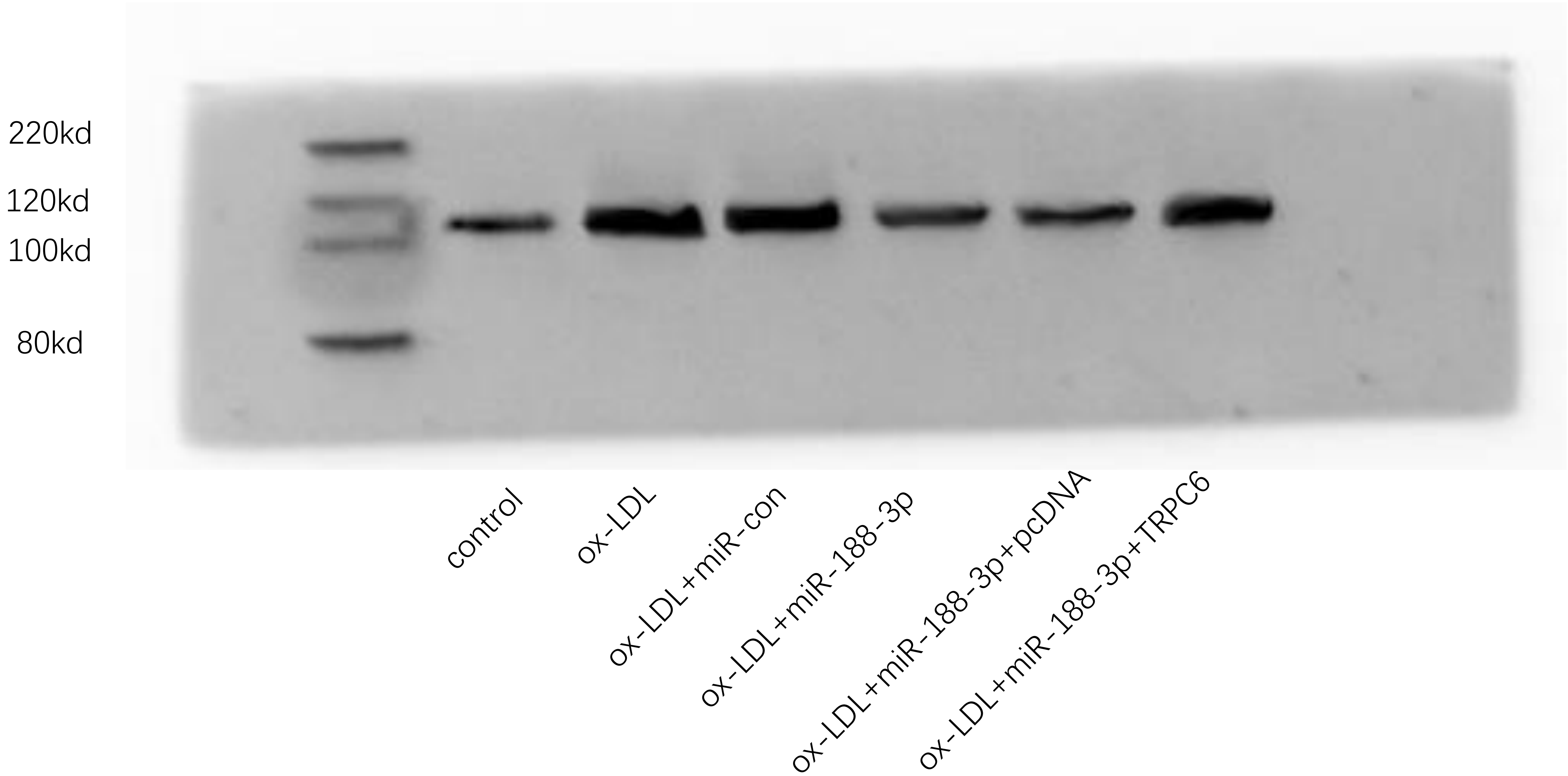
220kd
120kd
100kd
80kd
control
ox-LDL
ox-LDL+miR-con
ox-LDL+miR-188-3p
ox-LDL+miR-188-3p+pcDNA
ox-LDL+miR-188-3p+TRPC6

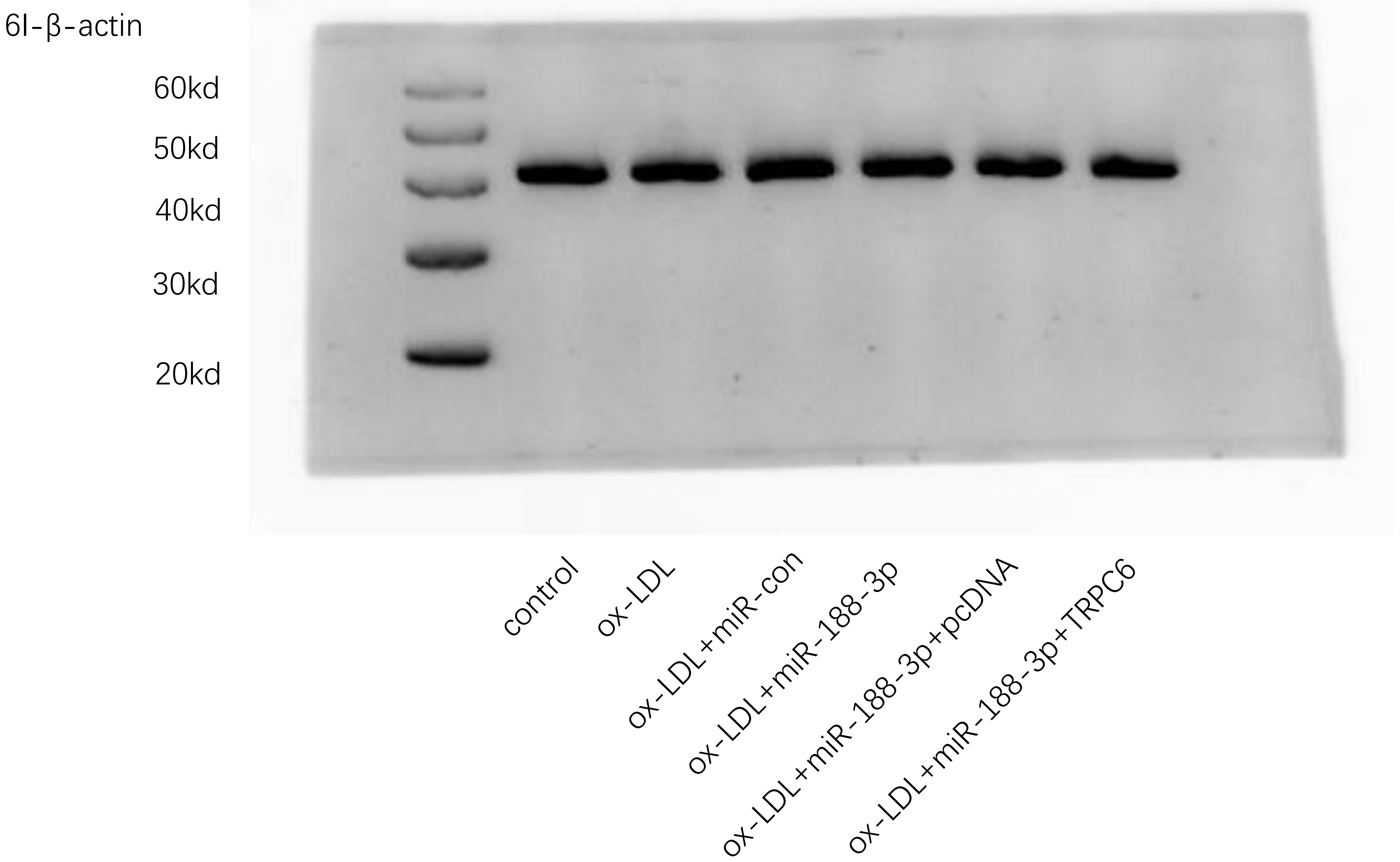
6I-β-actin
60kd
50kd
40kd
30kd
20kd
control
ox-LDL
ox-LDL+miR-con
ox-LDL+miR-188-3p
ox-LDL+miR-188-3p+pcDNA
ox-LDL+miR-188-3p+TRPC6

6I-BAX

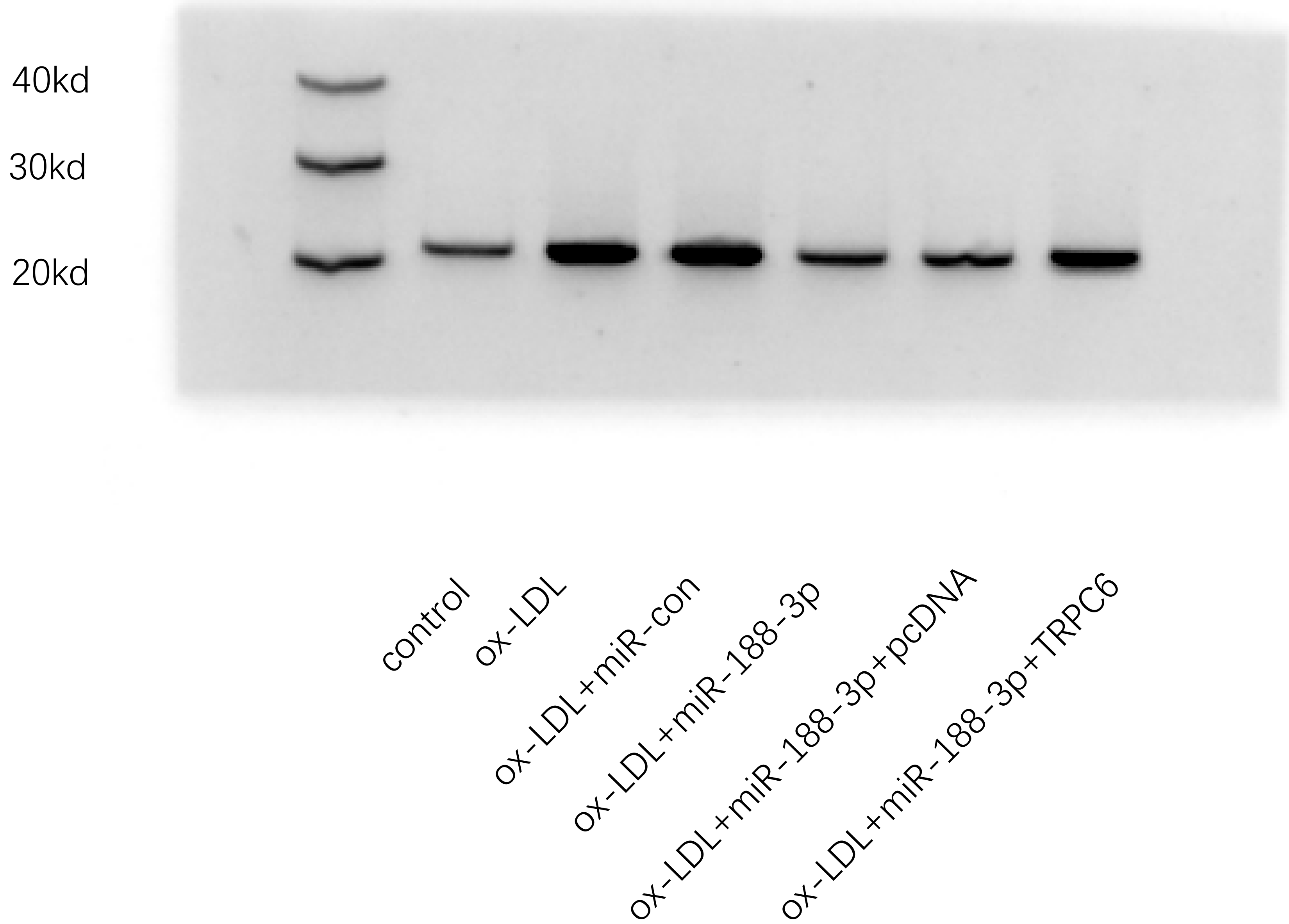
40kd
30kd
20kd
control
ox-LDL
ox-LDL+miR-con
ox-LDL+miR-188-3p
ox-LDL+miR-188-3p+pcDNA
ox-LDL+miR-188-3p+TRPC6

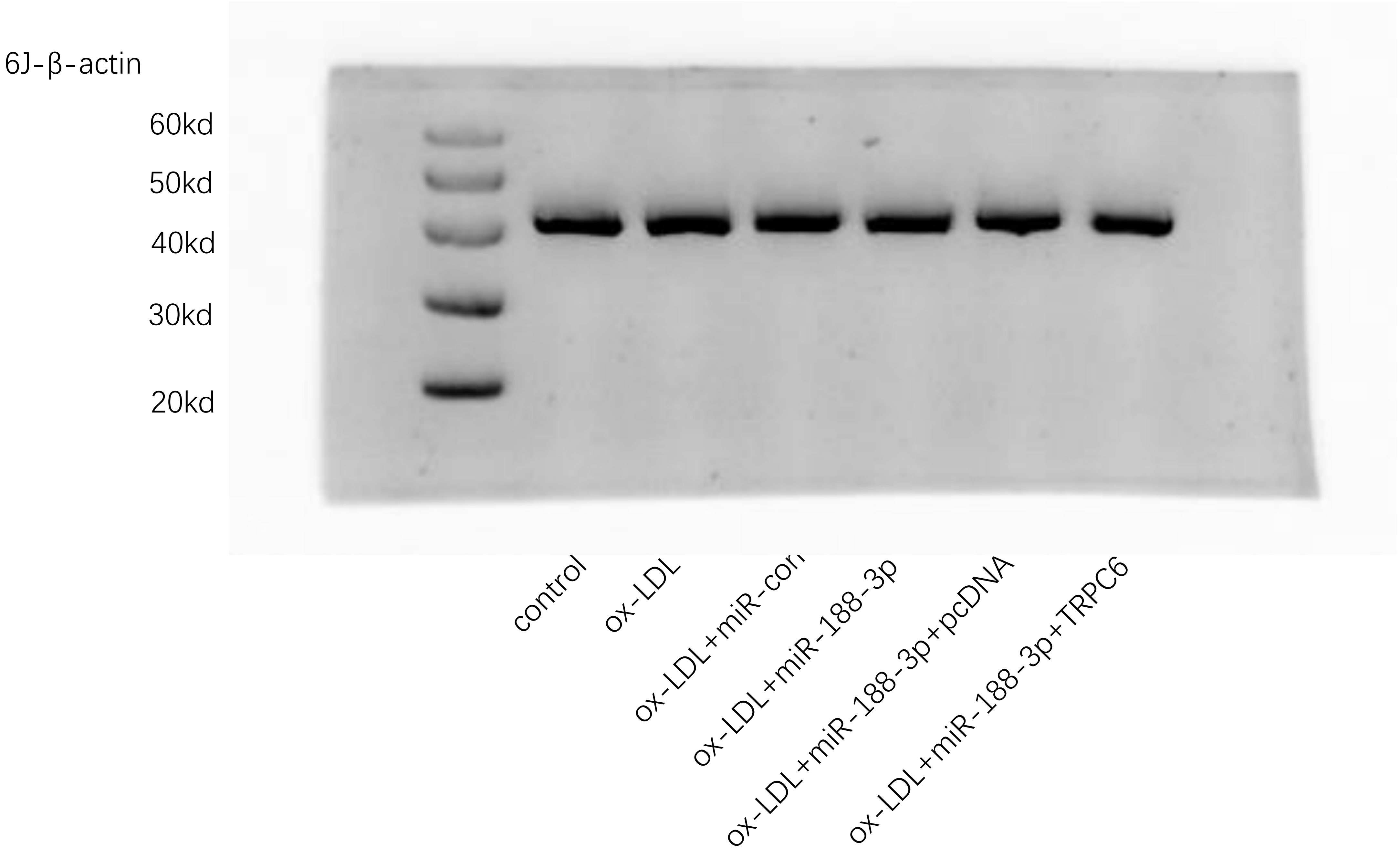

6J-β-actin
60kd
50kd
40kd
30kd
20kd
control
ox-LDL
ox-LDL+miR-con
ox-LDL+miR-188-3p
ox-LDL+miR-188-3p+pcDNA
ox-LDL+miR-188-3p+TRPC6

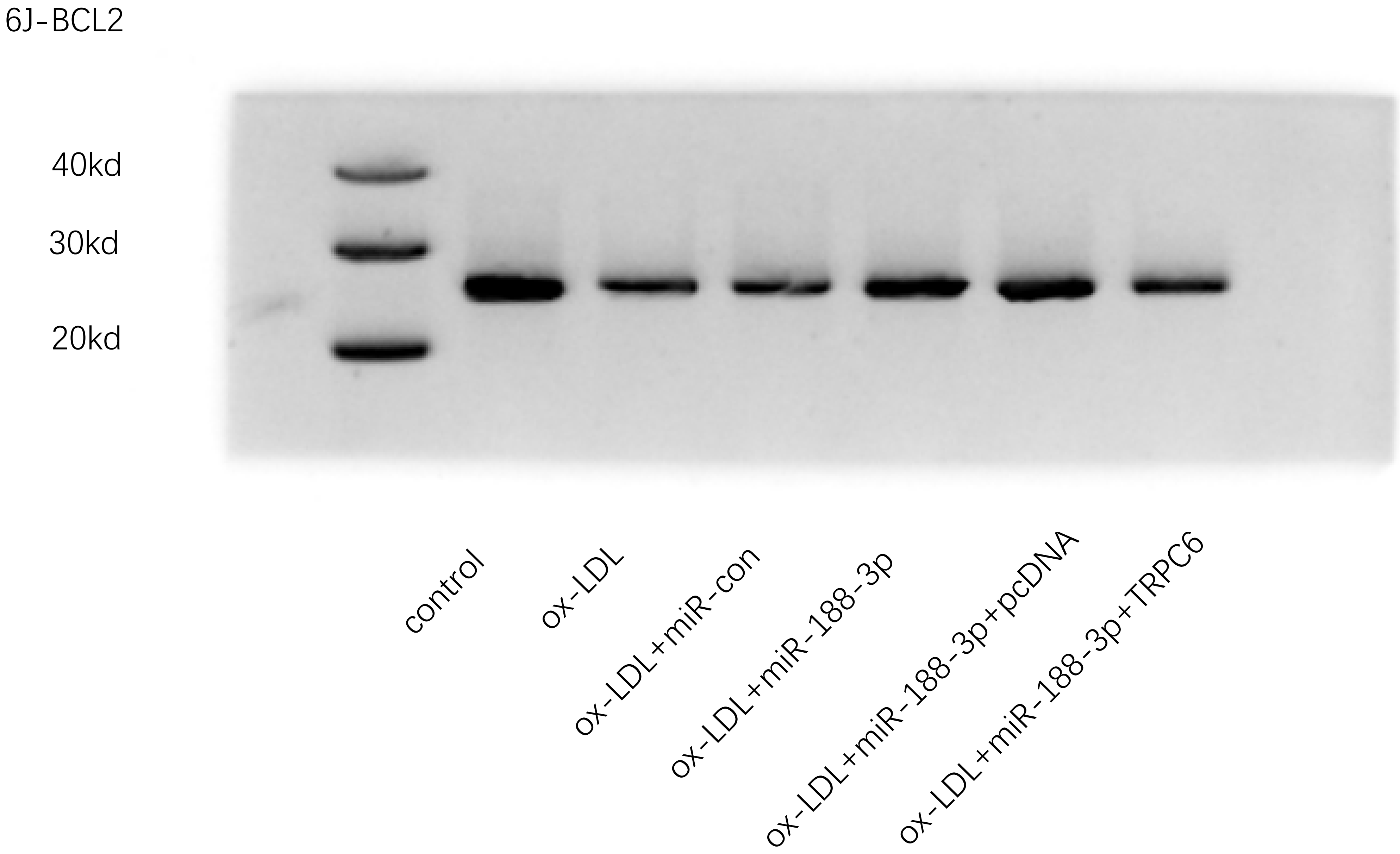
6J-BCL2
40kd
30kd
20kd
control
ox-LDL
ox-LDL+miR-con
ox-LDL+miR-188-3p
ox-LDL+miR-188-3p+pcDNA
ox-LDL+miR-188-3p+TRPC6
